# Current Approaches for Glioma Gene Therapy and Virotherapy

**DOI:** 10.3389/fnmol.2021.621831

**Published:** 2021-03-11

**Authors:** Kaushik Banerjee, Felipe J. Núñez, Santiago Haase, Brandon L. McClellan, Syed M. Faisal, Stephen V. Carney, Jin Yu, Mahmoud S. Alghamri, Antonela S. Asad, Alejandro J. Nicola Candia, Maria Luisa Varela, Marianela Candolfi, Pedro R. Lowenstein, Maria G. Castro

**Affiliations:** ^1^Department of Neurosurgery, University of Michigan Medical School, Ann Arbor, MI, United States; ^2^Department of Cell and Developmental Biology, University of Michigan Medical School, Ann Arbor, MI, United States; ^3^Laboratory of Molecular and Cellular Therapy, Fundación Instituto Leloir, Buenos Aires, Argentina; ^4^Immunology Graduate Program, University of Michigan Medical School, Ann Arbor, MI, United States; ^5^Cancer Biology Graduate Program, University of Michigan Medical School, Ann Arbor, MI, United States; ^6^Departamento de Biología e Histología, Facultad de Medicina, Universidad de Buenos Aires, Buenos Aires, Argentina

**Keywords:** gene therapy, glioma, viral vectors, non-viral vectors, HSV1-TK, mutant IDH1 3, immunotherapy, FMS-like tyrosine kinase 3 ligand

## Abstract

Glioblastoma (GBM) is the most common and aggressive primary brain tumor in the adult population and it carries a dismal prognosis. Inefficient drug delivery across the blood brain barrier (BBB), an immunosuppressive tumor microenvironment (TME) and development of drug resistance are key barriers to successful glioma treatment. Since gliomas occur through sequential acquisition of genetic alterations, gene therapy, which enables to modification of the genetic make-up of target cells, appears to be a promising approach to overcome the obstacles encountered by current therapeutic strategies. Gene therapy is a rapidly evolving field with the ultimate goal of achieving specific delivery of therapeutic molecules using either viral or non-viral delivery vehicles. Gene therapy can also be used to enhance immune responses to tumor antigens, reprogram the TME aiming at blocking glioma-mediated immunosuppression and normalize angiogenesis. Nano-particles-mediated gene therapy is currently being developed to overcome the BBB for glioma treatment. Another approach to enhance the anti-glioma efficacy is the implementation of viro-immunotherapy using oncolytic viruses, which are immunogenic. Oncolytic viruses kill tumor cells due to cancer cell-specific viral replication, and can also initiate an anti-tumor immunity. However, concerns still remain related to off target effects, and therapeutic and transduction efficiency. In this review, we describe the rationale and strategies as well as advantages and disadvantages of current gene therapy approaches against gliomas in clinical and preclinical studies. This includes different delivery systems comprising of viral, and non-viral delivery platforms along with suicide/prodrug, oncolytic, cytokine, and tumor suppressor-mediated gene therapy approaches. In addition, advances in glioma treatment through BBB-disruptive gene therapy and anti-EGFRvIII/VEGFR gene therapy are also discussed. Finally, we discuss the results of gene therapy-mediated human clinical trials for gliomas. In summary, we highlight the progress, prospects and remaining challenges of gene therapies aiming at broadening our understanding and highlighting the therapeutic arsenal for GBM.

## Introduction

### Molecular Alterations in Gliomas

Gliomas are group of heterogeneous primary brain neoplasms which differ in grade of malignancy, histology and genomic alterations. They may arise from neural stem cells (NSCs), NSC-derived astrocyte or oligodendrocyte precursor cells (Zong et al., 2012, 2015) and represent almost 30% of the central nervous system (CNS) tumors, and 80% of malignant CNS tumors (Ostrom et al., 2015, 2018). Most gliomas are diffuse and have been traditionally classified based either on histologic type: astrocytoma, oligodendroglioma, oligoastrocytoma (a rare mixed glioma) (Perry and Wesseling, 2016) or on their malignancy grade: World Health Organization (WHO), grades I-IV (Louis et al., 2007, 2016; Reifenberger et al., 2017). Recently, the WHO classification was refined (Louis et al., 2016). The presence and distribution of genetic alterations in brain tumors is now a criterion to differentiate glioma subtypes (Louis et al., 2016; Masui et al., 2016; Wesseling and Capper, 2018) that can be related with a particular WHO grade and tumor histology (Parsons et al., 2008; Sturm et al., 2012; Brennan et al., 2013; Cancer Genome Atlas Research et al., 2015; Ceccarelli et al., 2016) (Table 1).

**Table 1:** List of recurrent genomic alterations in glioma.

**Glioma subtype**	**mIDH1 LGG codel**	**mIDH1 LGG non-codel**	**mIDH1 secondary glioblastoma**	**wt-IDH1 adult glioblastoma**	**Diffuse midline glioma**	**Pediatric HGGs**	**Pediatric LGGs**
Grade	Diffuse glioma, WHO grade II/III	Diffuse glioma, WHO grade II/III	WHO grade IV	WHO grade IV	WHO grade IV	WHO grade IV	WHO grade I-III
Recurrent genetics alterations	IDH1, TERT, CIC, FUBP1, TCF12 Mutations; CDKN2A deletion	IDH1 or IDH2, TP53, ATRX mutations	IDH1 or IDH2, TP53, ATRX mutation; CDKN2A homozygous deletion	TERT, PTEN, TP53, PIK3CA, PIK3R1, NF1 mutation; PDGFRA amplification.	H3F3A-K27M or HIST1H3B/C-K27M, TP53, PPMD1, ACVR1, FGFR1 mutation, PDGFRA, MYC, MYCN, CDK4, CDK6, CCND1-3, ID2, MET amplification	H3F3A-G34, ATRX, TP53, DAXX mutation; EGFR amplification	BRAF-V600E mutation and KIAA1549:BRAF fusion, BRAF, RAF1, NTRK2 gene fusions; BRAF-V600E, NF1, KRAS, FGFR1 alterations
DNA/histone methylation	Histone and DNA Hyper-methylation; G-CIMP	Histone and DNA Hyper-methylation; G-CIMP	Histone and DNA Hyper-methylation; G-CIMP		Loss of histone-H3-lysine tri-methylation	Decreased H3K36me3 DNA hypo-methylation	
Chromosomal alterations	1p/19q co-deletion	loss of heterozygosity in 17p; 7q gain	loss of heterozygosity in 17p; 7q gain; 10q deletion	7q gain;			
Age	Young adults	Young adults	Adults	Adults	Children	Children	Children

A recurrent point mutation in isocitrate dehydrogenase 1 (IDH1), usually at arginine 132 (R132H), is detected with high frequency in adult diffuse-gliomas, being particularly high in diffuse low-grade gliomas (LGGs; WHO grade II) (Cancer Genome Atlas Research et al., 2015; Ceccarelli et al., 2016; Delgado-Lopez et al., 2017; Ostrom et al., 2018). This mutation is also found in anaplastic astrocytomas (WHO grade III), and in a smaller proportion of glioblastomas originated from LGGs (secondary glioblastomas; WHO grade IV) (Cancer Genome Atlas Research, 2008; Bai et al., 2016; Ceccarelli et al., 2016; Louis et al., 2016). Mutant IDH1 gliomas are sub-classified, according with the loss of 1p/19q chromosomal segments, in mutant IDH1-1p/19q-codel and mutant IDH1-noncodel. Mutant IDH1 1p/19q-codel gliomas frequently harbor *TERT* promoter (*TERT*p) and *CIC* mutations, and are associated with oligodendrogliomas; whereas mutant IDH1-non-codel harbor mutations in alpha-thalassemia X-linked mental retardation (*ATRX*) and *TP53*, and associated with astrocytoma and oligoastrocytoma (Cancer Genome Atlas Research et al., 2015; Bai et al., 2016; Ceccarelli et al., 2016; Louis et al., 2016; Venteicher et al., 2017).

In adults, wild-type IDH1 (wt-IDH1) glioma patients retain ATRX function, and typically present *TP53* and *TERT*p mutations, and alterations in regulators of the receptor tyrosine kinase (RTK)-RAS-PI3K signaling cascade, including EGFR amplification and *PTEN* mutation or loss (Brennan et al., 2013; Louis et al., 2016; Masui et al., 2016; Reifenberger et al., 2017). Pediatric gliomas are mostly wt-IDH1, and they also can harbor *TP53* and *ATRX* inactivating mutations, additionally mutations in *H3F3A, HIST1H3B, HIST1H3C*, and *BRAF* (Rapidly Accelerated Fibrosarcoma type B) are frequent in pediatric high-grade gliomas (HGGs) (Bjerke et al., 2013; Venteicher et al., 2017). Based on these alterations, four pediatric HGG subtypes can be distinguished: H3.3-K27M; H3.1-K27M, characteristic of high grade midline gliomas, including diffuse intrinsic pontine glioma (DIPG); H3.3G34-R/V; and BRAF-V600E (Jones et al., 2017). BRAF alterations are also found in pediatric LGGs (Packer et al., 2017).

In addition, DNA methylation in CpG islands describes the CpG-island methylator phenotype (G-CIMP) which is associated with better prognosis and tightly related with IDH1 mutation (Noushmehr et al., 2010; Wiestler et al., 2014). Recently, a study performed over more than 1,000 diffuse glioma (TCGA) patients, identified glioma DNA methylation clusters (LGm1–LGm6) which are linked to molecular glioma subtypes (Ceccarelli et al., 2016). Also, the methylation of CpG islands in the O6-methylguanine-DNA methyltransferase (MGMT) promoter has been identified as a molecular marker of better response to treatment with DNA alkylating agents (Wick et al., 2014).

The genetic lesions described in gliomas impact tumor biology and signaling pathways. Important signaling pathways altered in gliomas include the growth factor receptor tyrosine kinase (RTK) signaling pathways, partly as a result of PDGF and EGFR overexpression (Verhaak et al., 2010; Nazarenko et al., 2012). RAS, PI3K/PTEN/AKT, RB/CDK N2A-p16INK4a, and TP53/MDM2/MDM 4/CDKN2A-p14ARF pathways are commonly activated in gliomas and has been involved in cancer cells proliferation (Nakada et al., 2011; Crespo et al., 2015). In addition, NOTCH signaling activity has been reported in WHO grade IV gliomas, and can be associated with hypoxia, PI3K/AKT/mTOR and ERK/MAPK molecular pathways, increase malignant features of gliomas (Gersey et al., 2019).

In pediatric gliomas the MAPK pathway or its downstream effectors, which contribute to tumorigenesis and growth of many types of cancers, can be activated as a consequence of *NF1* and *BRAF* gene mutations (Truong and Nicolaides, 2015; Mackay et al., 2017) In addition, BMP signaling, is also active in pediatric HGG tumor cells (Mendez et al., 2020). Approximately 25% of childhood brainstem gliomas harbor somatic mutations in Activin A receptor type I (*ACVR1*) (Fontebasso et al., 2014) which encode the type I BMP receptor ALK2, inducing BMP pathway activation (Olsen et al., 2014). Signaling pathway alterations, resulting from specific genetic lesions in gliomas, represent a valuable target to develop novel targeted gene therapies.

### Barriers to Drug and Gene Delivery

One of the most challenging aspects in developing effective therapies for gliomas is the ability of the therapeutic agents to reach the tumor site at sufficient therapeutic concentrations (Shergalis et al., 2018). This is due to the presence of the Blood Brain Barrier (BBB), composed of a monolayer of endothelial cells held together by restrictive tight junctions (Vorbrodt and Dobrogowska, 2003). Pericytes, astrocytes, nerve terminals and central nervous system-border associated macrophages (BAMs), a specific myeloid subpopulation are closely associated with the endothelium and play critical roles in BBB development, maintenance and function (Abbott et al., 2010; Rajan et al., 2020). The BBB is a neuroprotective barrier that can block the passage of noxious agents but also the delivery of anti-tumor drugs including gene therapy delivery vehicles (Karim et al., 2016). Different strategies have emerged to offset these protective effects of the BBB, such as direct delivery of chemotherapeutics to the brain as well as the passive targeting based on the increased permeability and retention (EPR) effects (Yu et al., 2016). However, the passive targeting strategy is not sufficient to target invasive tumor cells, as the EPR effects tend to be weak near the infiltrating cancer cell tumor region (Juillerat-Jeanneret, 2008; Kim et al., 2015a). The blood-brain tumor barrier (BBTB), is also known to prevent drugs from accessing the tumor bulk, contributing to chemo-resistance and tumor recurrence. New strategies for actively targeting the BBB have been developed, such as disruptions in tight junctions (Karim et al., 2016), efflux transporter inhibition (Hoosain et al., 2015; Parrish et al., 2015) receptor-mediated transcytosis and/or endocytosis (Wei et al., 2014; Lajoie and Shusta, 2015).

Another consideration is the presence of P-glycoprotein efflux pumps that can actively transport lipophilic drugs out of the brain capillary endothelial cells that form the BBB. Although the BBB is altered at the tumor site, the dense endothelial cells' layer is not compromised and therefore the BBB remains effective at preventing drugs from reaching the tumor cells (Sarkaria et al., 2018). These issues need to be addressed during the preclinical phase, before bringing therapeutic candidates into clinical trials for brain cancer (Shergalis et al., 2018).

Due to the inefficient drug delivery across the BBB and development of drug resistance, gene therapy was envisioned as a promising strategy to overcome limitations of conventional therapies. Gene therapy for cancer treatment conventionally includes the introduction of growth regulating or tumor suppressor genes, RNA interference (RNAi) to inhibit the activity of oncogenes. It can also involve the delivery of suicide genes which can convert non-toxic prodrugs into active anti-cancer compounds. Other approaches include oncolytic and immunomodulatory gene therapy approaches (Candolfi et al., 2009; Puntel et al., 2010; Foreman et al., 2017; Mendez et al., 2020).

Delivery vectors such as viral vectors, non-polymeric nanoparticles (NPs) and polymeric NPs have been used to deliver the therapeutic payload in GBM and LGG (Caffery et al., 2019). To elicit therapeutic effects in the brain, nucleic acids used as therapeutic moieties need to surmount several barriers. Once they enter the blood circulation, they will encounter nuclease degradation, systemic elimination, reticuloendothelial system (RES) uptake before they can successfully cross the BBB, which is impermeable to hydrophilic macromolecules. After sufficient diffusion throughout the brain and into the tumor mass, the therapeutic gene needs to be endocytosed into targeted cells followed by endosome escape to avoid lysosomal degradation and eventually reach the cytoplasm for siRNA or further transport into the nucleus for plasmid DNA (Lu and Jiang, 2017). Viral vectors are attractive delivery vehicles, but they have not yet been clinically approved due in part to manufacturing challenges, high-cost, immunogenic and inflammatory responses, oncogenic mutations and limited loading capacity (Bergen et al., 2008; Rogers and Rush, 2012; Gomes et al., 2015). Non-viral delivery strategies offer alternative approaches that can be developed used to overcome the barriers of gene delivery. Many non-viral vectors, including polymeric and non-polymeric are non-immunogenic and can be functionalized with targeting moieties to increase receptor-mediated uptake of vectors into tumor tissue.

### Immune Responses in Glioma

Immunotherapy has proven successful against a growing number of tumors, unfortunately ongoing attempts to develop new immunotherapies for GBM have not yet demonstrated any significant improvement in glioma patients' survival. In Phase-III clinical trials, immune-checkpoint blockade immunotherapies, which looked highly promising in other solid cancers such as melanoma and lung cancer, were ineffective in GBM (Havel et al., 2019; Zhang et al., 2020). GBM exploits numerous strategies contributing to the evolution of an immunologically suppressive TME that eventually promotes systemic immunosuppression and antagonizes anti-GBM immune responses. GBM mediated immunosuppression is achieved by the production of cytokines and chemokines in the TME and subsequent recruitment of immunosuppressive cells, blocking intra-tumoral T-cell migration and activation (Perng and Lim, 2015). Systemic immunosuppression has been demonstrated by compromised adaptive immunity in murine GBM models and human subjects (Bloch et al., 2013; Chae et al., 2015). TGF-b and IL-10 play a central in maintaining the immunosuppressive TME, these cytokines are not only produced by GBM-infiltrating Tregs, but also by the GBM cells themselves (Perng and Lim, 2015). Another anti-inflammatory cytokine, IL-10 suppresses the activation and effector functions of DCs, macrophages and T cells, and inhibits the MHC-II expression in monocytes (Moore et al., 2001; Perng and Lim, 2015). Additionally, IL-10 promotes the expansion of myeloid-derived suppressor cells (MDSCs), Tregs (Tanikawa et al., 2012) and augments PD-L1 expression in monocytes and tumor-associated macrophages (TAMs) (Bloch et al., 2013). TGF-b is preferentially expressed in GBM cells, and involved in the blockade of T cell proliferation and activation in murine GBM models and human GBM patients (Bodmer et al., 1989). Higher TGF-b expression levels are correlated with poor prognosis and higher glioma grades (Zhang et al., 2014).

GBMs also produce large amounts of indolamine 2,3-dioxygenase (IDO) that triggers the recruitment of Tregs and suppresses effector T cells' activity by depleting tryptophan from the TME (Wainwright et al., 2012). GBM produces other immunosuppressive factors including colony stimulating factor 1 (CSF-1), NO, PGE2, Arg I, Gal-1, and VEGF (Nduom et al., 2015). PGE2 stimulates anti-inflammatory Th2 cytokines such as IL-4, Il-6, and IL-10 and suppress the production of Th1 cytokines. CSF-1 has been demonstrated to polarize the macrophages to M2 phenotype which enhances the glioma progression (Pyonteck et al., 2013). VEGF inhibits the DCs maturation and promotes angiogenesis (Gabrilovich et al., 1996). GBM derived chemokines CCL22 and CCL2 recruits Tregs which expresses CCR4 into the TME and blockade of these chemokines could improve antitumor immunity (Galvao and Zong, 2013).

Progression of GBM is dependent on the genetic lesions encountered within the tumor cells and also epigenetic alterations resulting in an immunosuppressive glioma microenvironment. Immunosuppressive cells abundant within the glioma microenvironment include of MDSCs (Kamran et al., 2017; Guo et al., 2018), TAMs (Wei et al., 2020), and Tregs (Chang et al., 2016). MDSCs have been shown to promote tumor angiogenesis *via* secretion of VEGF as well as MMP-9, and also augment the expression of checkpoint receptor ligand PD-L1 (Mirghorbani et al., 2013). We have recently demonstrated that depletion of MDSCs in glioma-bearing mice prominently augments the efficacy of our immune stimulatory gene therapy (Kamran et al., 2017). Immunotherapeutic strategies currently being investigated to treat GBM include passive immunotherapy with antibodies (Kamran et al., 2016), chimeric antigen receptor (CAR) T-cell therapy (Pituch et al., 2018; Choi et al., 2019) autologous activated lymphocytes therapy (Walker et al., 2019; Lee-Chang et al., 2021), immune-mediated gene therapy (Ali et al., 2005; Curtin et al., 2009; Mineharu et al., 2012; Kamran et al., 2017), oncolytic viral therapy (Mooney et al., 2019; Chastkofsky et al., 2020), or active immunotherapy with tumor cell based vaccines, peptides, or dendritic cells (Hdeib and Sloan, 2015; Polivka et al., 2017).

#### T-cell Exhaustion, TAMs, MDSCs, Tregs

In glioma, most of the macrophages found within the tumor microenvironment have immune suppressive functionality and support tumor progression (Hambardzumyan et al., 2016). This population of tumor associated macrophages, TAMs, can constitute up to one-third the total mass of the tumor (Roesch et al., 2018). Brain-resident microglial cells and bone marrow derived macrophages (BMDMs) are distinct myeloid cell populations with many shared features including their immunoregulatory abilities and many surface markers (Roesch et al., 2018). Distinguishing features of these populations are that naïve-mature microglia expresses CD45^lo/int^ and BMDMs express CD45^hi^ (Roesch et al., 2018).

MDSCs play a major immune suppressive role in the TME and are correlated with glioma progression and therapeutic resistance (Kamran et al., 2018b; Ostrand-Rosenberg and Fenselau, 2018). MDSCs are divided into two main subpopulations polymorphonuclear-MDSCs (PMN-MDSCs) and monocytic-MDSCs (M-MDSCs) (Kamran et al., 2018b). They are characterized by different sets of surface markers (Kamran et al., 2018b). MDSCs in GBM have also been found to express high levels of the T-cell exhaustion promoting molecule PD-L1 (Kumar et al., 2016). Study revealed that immune suppressive cells work, in part, by inducing T cell exhaustion. Many T cells within the TME of GBM exhibit an exhausted T cell phenotype, with lower secretion of IFN-γ, IL-2, and TNF-α (Woroniecka and Fecci, 2018; Woroniecka et al., 2018). In addition, exhausted T cells may highly express multiple “inhibitory” receptors, including PD-1, 2B4 (CD244), BTLA, CTLA-4, CD160, LAG-3, and Tim-3 (Wherry and Kurachi, 2015; Osuch et al., 2020). Currently, therapies targeting the classical immune checkpoint pathways responsible for inducing the exhausted T cell phenotype, PD-1 to PD-L1 and CD80/CD86 to CTLA4, are being used to reverse the dysfunctional state and enhance anti-tumor immune response (Kamran et al., 2017; Woroniecka and Fecci, 2018; Woroniecka et al., 2018). Although blocking the immune checkpoints with anti-PD-1, anti-PD-L1 and anti-CTLA4 has shown promising results and is an effective strategy for many other types of cancers, their ability to bolster the immune response is limited in the case of GBM (Woroniecka and Fecci, 2018; Woroniecka et al., 2018). High frequencies of Tregs are found in gliomas and this occurrence has also been associated with tumor progression and immune evasion (Mu et al., 2017). Tumor cells recruit Tregs by the CCL22/CCR4 and CCL28/CCR10 signaling axes in GBM. IDO expression on GBM tumor cells has also been shown to stimulate Treg recruitment (Mu et al., 2017).

## Gene Therapy and Virotherapy in Glioma

Gene therapy is a therapeutic approach that consists in utilizing genetic elements in order to treat or prevent disease. Whole genes, regulatory elements or oligonucleotides may be delivered to the target cells in glioma patients either by mechanical methods or using delivery vehicles. In order to achieve high therapeutic efficacy, gene therapy vectors must be chosen with caution, taking into consideration therapeutic transgene expression levels, distribution of gene expression within the TME, immunogenicity and biosafety (Castro et al., 2014; Asad et al., 2017; Kamran et al., 2018a). Gene therapy viral and non-viral vectors have shown efficacy in many pre-clinical studies since their first development in the 90s (Okura et al., 2014), but their clinical implementation still presents many challenges (Lowenstein et al., 2009), which we will highlight below. One of the advantages of gene therapy is that its local administration may overcome the challenges posed by the BBB for systemic delivery approaches. Virotherapy is also an attractive therapeutic approach for glioma; it entails the use of genetically engineered viruses, which are no longer virulent and thus, cannot cause disease, but have the capacity of replicating within tumor cells, causing tumor cell death and release of oncolytic viral particles which can continue to infect and kill neighboring tumor cells.

### Viral Vectors for Gene Therapy

#### Adenoviral Vectors

Adenoviruses are non-enveloped, double stranded DNA viruses that exhibit many advantages i.e., feasibility for genetic manipulation, high titers, low biosafety risks, and excellent safety profile after delivery into the brain. They are able to transduce dividing and non-dividing cells, while their genome remains episomal, thus reducing the risk of insertional mutagenesis. Adenoviral vectors (AdV) genome consists of ~35 kbp. They possess high cell tropism, since AdV are able to bind to the target cells *via* the interaction between their knob domain and the coxsackie and adenovirus receptor (Castro et al., 2014). AdV can also enter cells by endocytosis after interacting with cell surface integrins (Castro et al., 2014).

First-generation AdV were initially produced by deletion of E1 and E3 regions, which makes them non-replicative (Castro et al., 2014) and are replaced by the expression cassette, which could be up to 8 kbp. Several strategies have been developed for GBM using AdVs. AdVs encoding for the conditionally cytotoxic enzyme Herpes Simplex Virus thymidine kinase (HSV-TK) has exhibited very promising results in clinical trials in GBM. Expression of HSV-TK in glioma cells confers sensitivity to ganciclovir (GCV), as explained below (van Putten et al., 2010). An interesting immunotherapeutic strategy involved the development of a dendritic cell (DC)-specific AdV that targets DEC205, a DC surface receptor, expressing human glioma-specific antigen (CMV-IE) (Kim et al., 2018). This approach showed prolonged survival in a GBM model and, when rechallenged, brain tumor cells were completely rejected (Kim et al., 2018). Since AdV are not completely devoid of viral genes, they are immunogenic, which leads to transient transgene expression (Barcia et al., 2007).

Adeno-associated viruses (AAV) are small replication-defective non-enveloped single stranded DNA viruses from the non-pathogenic parvovirus family (Asad et al., 2017). AAV require a helper virus for its replication inside the host cell, such as adenovirus or HSV (van Putten et al., 2010). AAV have many advantages (Santiago-Ortiz and Schaffer, 2016) and among them AAV have a genome of 4.7 kbp, allows them to rapidly penetrate solid tumors, such as gliomas (Enger et al., 2002). It was reported that a single intracranial injection of AAV encoding human interferon (IFN)-β in human and murine GBM models increases tumor cell death and promotes long-term survival (GuhaSarkar et al., 2017). Many researchers have developed high-efficiency AAV for GBM cells, by selection in culture of a chimeric AAV capsid library generated by DNA shuffling of different *cap* genes, with several different AAV serotypes (Maguire et al., 2010; Zolotukhin et al., 2013). Despite of the many advantages of this vector, at the moment they are not being evaluated in clinical trials; this should be expected soon.

#### Retroviral Vectors

Retroviruses are single stranded positive sense RNA viruses, whose RNA genome is reverse transcribed into DNA that integrates into the genome of the host cell (Murphy and Rabkin, 2013). They have a cloning capacity of ~8 kbp, with stable expression of the therapeutic transgene, and can only infect dividing cells (Murphy and Rabkin, 2013). Retroviral vectors (RV) encoding HSV-TK were the first viral vectors to be evaluated in clinical trials for glioma (NCT00001328). This study showed anti-tumor activity, but only in smaller tumors (Caffery et al., 2019). A tumor-selective non-lytic replicating RV, Toca 511, and an extended-release formulation of 5-fluorocytosine (5-FC), Toca FC, enables highly efficient transduction of glioma cells with cytosine deaminase (CD), an enzyme that activates the conversion of 5-FC into the anticancer drug 5-fluorouracil (5-FU) directly within the infected cells (Takahashi et al., 2014). Researchers showed that this treatment also sensitizes GBM cells to radiotherapy (Takahashi et al., 2014). A previous study also revealed tumor eradication and prolonged survival in immunocompetent mice (Ostertag et al., 2012).

#### Lentiviral Vectors

Lentiviruses are single stranded positive sense RNA viruses that have been widely evaluated for the treatment of GBM (Del Vecchio et al., 2019). They are similar to RV but exhibit several advantages, mostly because lentiviral vectors (LV) integrate into the host genome but are less prone to insertional mutagenesis. The best-known lentivirus is the human immunodeficiency virus type (HIV)-1, which in 1994 was first seen to transduce lymphocytes (Parolin et al., 1994) and non-dividing cells (Naldini et al., 1996). Third generation HIV-based vectors have been developed with higher transduction efficiency and safety. These vectors may be modified in order to achieve tissue tropism by pseudotyping and exhibit low immunogenicity due to the lack of viral protein expression (Del Vecchio et al., 2019). Lymphocytic choriomeningitis virus-pseudotyped LV were developed to achieve higher transduction efficiency in GBM cells, including glioma stem cells, in relation to normal brain cells (Miletic et al., 2004; Huszthy et al., 2009). LV are the vectors of choice to express silencing RNA (Luan et al., 2015) or for engineering T cells so that they express chimeric antigen receptors specific for GBM antigens (Yu et al., 2017). Researchers developed a LV with a p2A peptide-enabled dual expression system allowing the expression of tumor suppressor proteins growth arrest specific (GAS)-1 and PTEN under the control of a CMV promoter (Sanchez-Hernandez et al., 2018). This vector inhibited the growth of human GBM cells *in vitro* and elicited inhibition of glioma progression in a human GBM xenograft model (Sanchez-Hernandez et al., 2018). A LV encoding a shRNA specific for TLX, an orphan nuclear receptor (NR2E1), essential for neural-stem cell renewal, inhibited human glioma stem cell tumorigenicity in mice, and induced the expression of DNA hydroxylase ten eleven translocation 3 (TET3), a potent tumor suppressor downstream of TLX (Cui et al., 2016). LVs have also been used to encode the Clustered Regularly Interspaced Short Palindromic Repeats (CRISPR) and CRISPR-associated (Cas) 9 system. Using this system, it was reported that TEA domain transcription factor 1 (TEAD1) ablation inhibited human GBM cell migration and altered the migratory and epithelial mesenchymal transition (EMT) transcriptome signatures (Tome-Garcia et al., 2018).

### Non-viral Vectors for Gene Therapy

Non-viral vectors are emerging as attractive platforms for gene therapy approaches for GBM. Recent studies discussed below, have demonstrated the potential of these delivery technologies.

#### Non-polymeric Delivery System

##### Liposomes

Liposomes are artificial, lipid-based microvesicles that are considered as a possible valuable system to achieve therapeutic efficacy in glioma. On this backdrop, a liposomal vector was devised in early 2000s to carry a plasmid coding for HSV-TK which was given to patients with recurrent GBM in a Phase I/II trial *via* intratumoral infusion, followed by administration of the prodrug ganciclovir (Reszka et al., 2005). This therapy was well-tolerated without major side effects. Also, they observed >50% reduction of tumor volume in patients. Although this was a small Phase I trial and thus, it was not powered to determine therapeutic efficacy. Moreover, Kato et al. demonstrated that siRNA-based downregulation of MGMT could enhance the chemosensitivity of malignant gliomas against TMZ using novel liposome, LipoTrust EX Oligo. Such liposome transduced glioma cells are found to be sensitized to TMZ both *in vivo* and *in vitro* models (Kato et al., 2010). A dual targeting with T7 and A7R peptides was developed to target vascular endothelial growth factor receptors 2 (VEGFR2) (Zhang et al., 2017). PEG-conjugated liposomes modified with the Transferrin receptor (TfR) monoclonal antibody (OX26) and chlorotoxin (CTX) significantly promoted cell transfection, increased the transport of plasmid DNA bearing hTERTC27 gene across the BBB and efficiently targeted brain glioma cells both *in vitro* and *in vivo*. This dual targeting therapeutic strategy of OX26/CTX-pL/pC27 against glioma exhibits significant therapeutic efficacy leading to diminished tumor volume and extended survival of glioma bearing rats (Yue et al., 2014). Other liposomal formulations with modified surface and core include magnetite-core cationic liposomes that can be used to activate a heat-shock sensitive promoter in the DNA carried by the liposome, thus regulating expression of the therapeutic gene such as TNFα in glioma cells (Ito et al., 2000).

##### Nanoparticles

NU-0129 is a spherical nucleic acid gold nanoparticle containing siRNAs targeting Bcl-2-like protein 12 (Bcl2L12) is now in early phase I clinical trials (NCT03020017) for patients with recurrent glioblastoma. It can cross BBB in xenograft GBM mice after systemic administration which results in increased apoptosis of glioma cells and reduced tumor progression (Jensen et al., 2013). RNA nanoparticles are also used to deliver anti-miR-21 in xenograft GBM mice, resulting in tumor regression and increased survival (Lee et al., 2017). Intravenously-administered chlorotoxin (CTX) coupled stable nucleic acid lipid particle (SNALP) formulated anti-miR21 oligo preferentially accumulates within the brain tumor and efficiently silence miR21 expression. This results in increased mRNA and protein levels of RhoB, leading to reduced tumor load and proliferation without inducing any systemic immunogenicity (Costa et al., 2015). Moreover, combined treatment of both nanoparticles formulated anti-miR21 oligo and tyrosine kinase inhibitor Sunitinib exerts enhanced apoptosis and improved survival in mice (Costa et al., 2015).

Development of a library with PBAE based nanoparticles carrying herpes simplex virus type I thymidine kinase (HSV-TK) DNA, resulted in apoptosis of transfected glioma cells. This led to increased median survival of glioma bearing animals when delivered intracranially (Choi et al., 2020). Furthermore, when HSV-TK DNA loaded nanoparticles are delivered in combination with the prodrug ganciclovir (GCV) to glioma cells *in vivo*, they elicited induction of apoptosis and reduction of tumor load in glioma bearing rats (Mangraviti et al., 2015). Another important type of anti-GBM treatment in gene therapy uses different types of RNA such as dsRNA, siRNA or miR101 associated to nanoparticulate systems resulting in enhanced apoptosis of GBM cells. Also inhibition of growth and migration of these cells can be induced through targeting miR34 or proteins like SOX9 and Ras with the same nanoparticulate systems (Shu et al., 2014; Kim et al., 2015b; Alphandery, 2020).

We have recently demonstrated that local treatment of glioma with sHDL (synthetic High-density lipoprotein) mimicking nanodiscs containing ApoAI mimetic peptide, phospholipids, immunogenic cell death inducing chemotherapeutics (ICD) docetaxel and adjuvant CpG oligodeoxynucleotide, effectively elicit anti-glioma T-cell activity and induce immunological memory response against tumor relapse (Kadiyala et al., 2019). We also engineered an albumin based NPs equipped with cell-penetrating iRGD peptide, containing siRNA against Signal Transducer and Activation of Transcription 3 factor (STAT3*i*) and demonstrated that when administered in combination with ionizing radiation, these NPs activate anti-GBM immunologic memory which results in tumor regression and long term survival of GBM bearing mice (Gregory et al., 2020). Other peptide modifications on nano-platforms have been explored to minimize off-target accumulation and facilitate active tumor targeting or mediate BBB transport. For example, IL-13Rα2 is overexpressed on glioma cells, therefore it is an attractive target for peptide-modified nanotherapies (Madhankumar et al., 2006). A study revealed that IL-13-conjugated nanoplatform enhanced therapeutic efficacy in a subcutaneous mouse model of glioma (Madhankumar et al., 2006). Moreover, transferrin receptor (TfR) has been extensively researched as a target for gliomas, because TfR is over-expressed on glioma cells (Kang et al., 2015). Despite exploiting the use of TfR as a target for decades, translation of systems leveraging these finding have been limited (Johnsen et al., 2019). On this backdrop, a seven amino acid peptide (sequence: HAIYPRH, T7), which has a greater affinity for TfR has been used for glioma targeting to deliver siRNA (Wei et al., 2016), coupled with other targeting ligands to demonstrate increased transport across the BBB and greater tumor penetration (Zong et al., 2014).

#### Oncolytic Viruses

**Figure 1:**
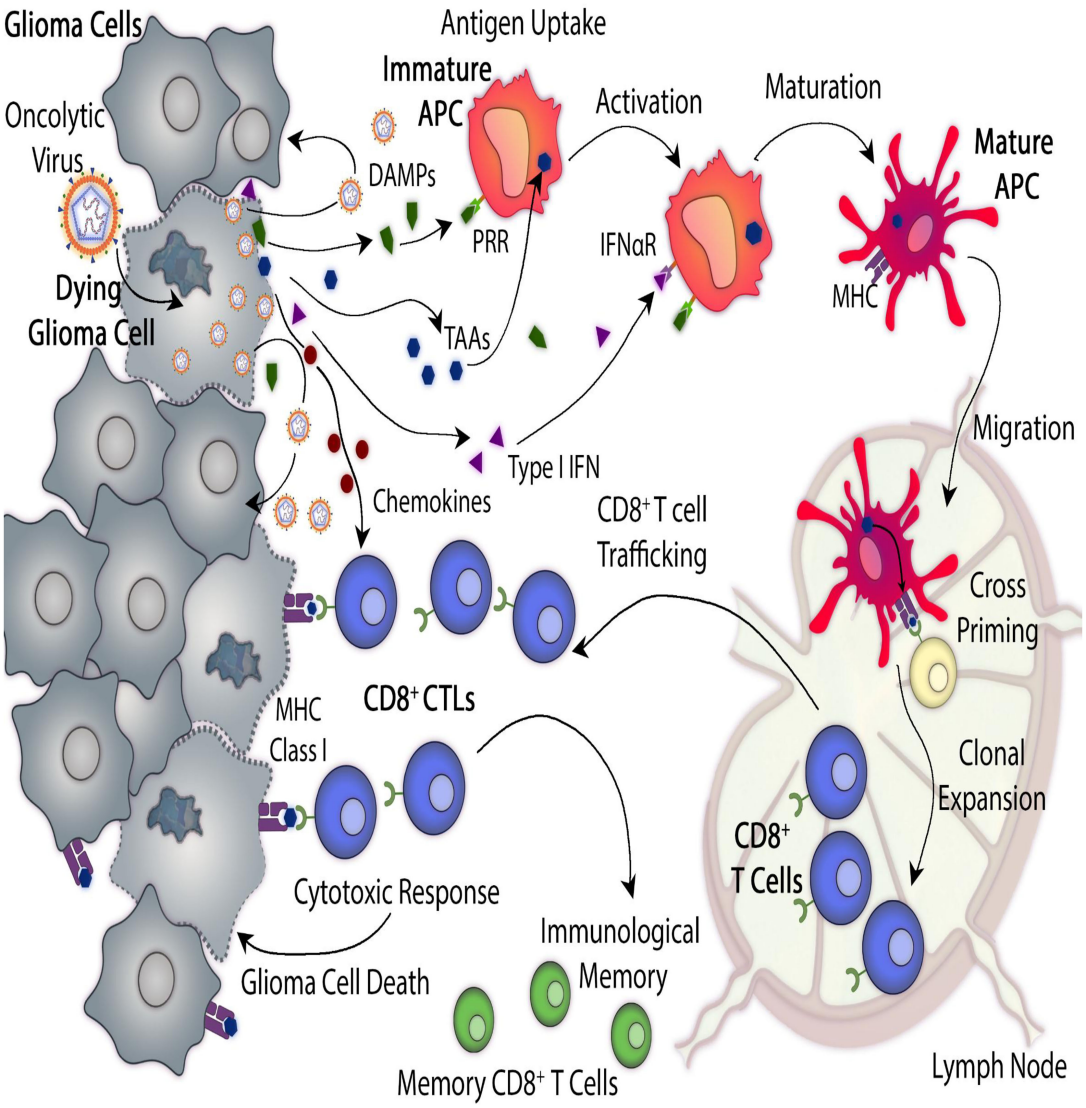
Antitumor mechanisms mediated by oncolytic virus-mediated therapy. Oncolytic viruses (OVs) induce glioma cell death by infecting cells and replicating within them. In addition, OVs trigger immunogenic cell death (ICD) which leads to anti-glioma immunity. Direct virus-mediated cell lysis induces the release of additional virus particles which can infect neighboring glioma cells and continue their replicative cycle. ICD produces immune stimulatory molecules such as tumor cells-derived damage-associated molecular patterns (DAMPs), chemokines and type I interferons (Type I IFN) and they also induce the release of tumor-associated antigens (TAAs). These molecules recruit antigen-presenting cells (APCs) to the site of viral infection, where they get activated, they engulf TAAs and recognize DAMPs which interact with their pattern recognition receptors (PRRs). Mature APCs migrate to the regional lymph node where they prime anti-tumor cytotoxic CD8^+^ T lymphocytes (CTLs) which leads to anti-glioma immunity. Viral-mediated release of type I IFN and chemokine elicits the recruitment of tumor-specific CTLs to the tumor site. As glioma cells express TAAs, presented by their major histocompatibility complex (MHC) class I, they are recognized, and therefore killed, by CD8^+^ T cells.

Several oncolytic viruses have been evaluated in preclinical studies or clinical trials for the treatment of GBM. Specificity must be seriously evaluated, taking into consideration the infection capacity of the vector. Oncolytic viruses (OVs) are designed to recognize tumor receptors or to replicate under oncogene promoters in order to improve their tropism and avoid non-neoplastic cells. It was observed that the immunosuppression present in the tumor microenvironment promotes the OV infection capacity and improves the oncolysis (Tobias et al., 2013; Davola and Mossman, 2019). Once infected, the dying tumor cells start the presentation of tumor epitopes, triggering a viral-specific and tumor-specific T cell-mediated immune response, critical for the efficiency of the oncolytic virotherapy (Li et al., 2017). When tumor cells are lysed, tumor-associated antigens (TAA) are released into the tumor microenvironment and recognized by the immune system, which stimulates the recruitment of activated immune cells which overcome the tumor-mediated immunosuppression and activate a systemic response (Figure 1) (Marelli et al., 2018). When using antitumor viral gene therapy, the administration and distribution of the vectors must be evaluated, taking into consideration their ability to overcome antiviral immune responses and to cross the BBB.

A genetically engineered third generation oncolytic HSV, G47Δ that is armed with IL-12 showed increased survival in a syngeneic murine GBM stem cell model (Cheema et al., 2013). G47Δ was evaluated in a phase II clinical trial in patients with GBM, who received repeated intratumoral stereotactic injections, in addition to TMZ (Todo, 2019).

Newcastle disease virus (NDV) based vectors have a natural tropism for tumor cells, together with oncolytic potential and immuno-stimulatory properties (Schirrmacher et al., 2019). It was shown that the complementary treatment with LaSota strain of the naturally oncolytic NDV induces increased apoptosis in glioma cells, comparing with TMZ alone (Bai et al., 2018). The combination treatment also significantly extended survival in a rat xenograft tumor model (Bai et al., 2018).

Finally, *in vivo* immunovirotherapy with measles virus (MV) strains in combination with anti-PD-L1 blockade synergistically increased the survival of a murine syngeneic GBM model, together with the enhanced infiltration of activated CD8^+^ T cells (Hardcastle et al., 2017). MV has already been evaluated in a dose-escalating phase I clinical trial in recurrent GBM in which no dose limiting toxicities were observed (NCT00390299) (**Table 3**).

## Approaches to Gene Therapy

This section was structured taking into account the gene therapeutic approaches against glioma (Table 2) that are currently under Phase-I/II/III clinical trials (Table 3). We will discuss advantages and limitations of the proposed approaches. We have included the clinical trials that were listed, in clinicaltrials.gov using the key words: “Condition or disease: glioma;” “Study type: interventional studies (clinical trials)”; “Status: Recruiting, not recruiting, not yet recruiting, and active;” “Phase: Phase 1, Phase 2, Phase 3.” For “other terms” we used the following words: gene therapy, virus, and antibody. Table 3 was updated in October 2020 and includes all the clinical trials found under those key words. Trials were organized depending upon 10 major viral vectors currently used in clinical trials.

**Table 2:** List of Viral vectors used in glioma gene therapy.

**Vectors**	**Genome**	**Advantages**	**Disadvantages**
Adenovirus (AdV)	Non-enveloped dsDNA Cloning capacity: 8 kbp	Production in high titers. Transduction of diving and non-dividing cells. Non-integrative, avoids insertional mutagenesis. Replicative or oncolytic. Transduce wide varieties of cells. Feasibility for being safely manipulated. Robust expression.	Highly Immunogenic. Existence of anti-AdV immunity in the population, leading to the clearance of the vector.
Adeno-associated virus (AAV)	Non-enveloped dsDNA Cloning capacity: 8 kbp Parvovirus family	Helper virus-dependent replication. They remain episomal in the absence of helper virus. Transduce dividing and non-dividing cells. Lack of immunogenicity Its small size allows the penetration into solid tumors. Transduce wide varieties of cells. Long-term expression.	Possibility of insertional mutagenesis. Low transduction in certain cell types. Existence of anti-AdV immunity in the population, leading to the clearance of the vector. Small cloning capacity.
Retrovirus (RV)	Enveloped ss(+)DNA Cloning capacity: 8 kbp	Stable transgene expression. Feasibility to be modified to achieve higher tropism.	Possibility of inserional mutagenesis. Infection of dividing cells only. Production in low titers Their manipulation requires more biosafety.
Lentivirus (LV)	Enveloped ss(+)DNA Cloning capacity: 8 kbp	Transduce dividing and non-dividing cells. Stable transgene expression. Their integration is less prone to insertional mutagenesis than RV. Transduce hematopoietic cells. Feasibility for being engineered to avoid integration, increasing biosafety. Feasibility to be modified to achieve higher tropism	Possibility of insertional mutagenesis. Engineered non-integrative LV have less stable transgene expression. Production in low titers. Their manipulation requires more biosafety.
Baculovirus (BV)	Enveloped dsDNA Cloning capacity: 38 kbp	Non-integrative, avoids insertional mutagenesis. They do not replicate in human cells, which makes them very safe. There is no pre-existent anti-BV immunity in the population. Transduction of wide varieties of cells. Feasibility to be modified to achieve higher tropism. Large cloning capacity.	They have not been evaluated in clinical trials. Unstable long-term storage.
Herpes simplex virus (HSV)	Enveloped dsDNA Cloning capacity: 20 kbp	Non-integrative, avoids insertional mutagenesis. Replicative or oncolytic. Feasibility to be modified to achieve higher tropism. Large cloning capacity.	Pathogen to humans, so they must be engineered. Immunogenicity. Existence of anti-HSV immunity in the population, leading to the clearance of the vector. Production in low titers.
Newcastle disease virus (NDV)	Enveloped ss(-)RNA Paramyxoviridae family	Non-pathogen to humans. High tropism Oncolytic Selective replication in tumor cells	Limited gene insertion. Limited manipulation.
Measles virus (MV)	Enveloped ss(-)RNA Paramyxoviridae family	High tropism. Feasibility to be modified for being retargeted. Oncolytic Selective replication in tumor cells.	Pathogen to humans, so attenuated strains must be used. Limited gene insertion.

**Table 3:** List of Viral vectors under clinical trials for glioma.

**Sr. no**.	**Viral vector**	**Gene therapy**	**Combination therapy**	**Condition**	**Phase**	**Clinical trial**	**Status**	**ID**
1	AdV	HSV-TK	Valacyclovir + Std treatment	GBM, Anaplastic astrocytoma	I-II	HSV-tk and XRT and chemotherapy for newly diagnosed GBM	Recruiting	NCT03603405
2		HSV-TK	Valacyclovir + radiation	Recurrent GBM, Astrocytoma grade III	I-II	HSV-tk + Valacyclovir + SBRT + chemotherapy for recurrent GBM	Recruiting	NCT03596086
3		AdV-TK	Ganciclovir + chemotherapy	High grade glioma	II	ADV-TK Improves outcome of recurrent high-grade glioma	Completed	NCT00870181
4		HSV-TK	Valacyclovir + radiation	Malignant glioma, GBM, Anaplastic astrocytoma	Ib	Phase 1b study of AdV-tk + Valacyclovir combined with radiation therapy for malignant gliomas	Completed	NCT00751270
5		HSV-TK	Valacyclovir + Std treatment	Malignant glioma, GBM, Anaplastic astrocytoma	IIa	Phase 2a study of AdV-tk with standard radiation therapy for malignant glioma (BrTK02)	Completed	NCT00589875
6		HSV-TK + Flt3L		Malignant glioma, GBM	I	Combined cytotoxic and immune-stimulatory therapy for glioma	Active	NCT01811992
7		HSV-TK	Ganciclovir + Chemotherapy	Brain and CNS tumors	I	Gene therapy in treating patients with primary brain tumors	Completed	NCT00002824
8		p53		Brain and CNS tumors	I	Gene therapy in treating patients with recurrent malignant gliomas	Completed	NCT00004041
9		p53		Brain and CNS tumors	I	Gene therapy in treating patients with recurrent or progressive brain tumors	Completed	NCT00004080
10		AdV-TK	Valacyclovir + Radiation	Pedriatic brain tumors including GBM, anaplastic astrocytoma, recurrent ependymomas	I	A phase I study of AdV-tk + prodrug therapy in combination with radiation therapy for pediatric brain tumors	Active	NCT00634231
11		Delta-24-RGD (oncolytic AdV)		Recurrent GBM	I-II	Safety study of replication-competent adenovirus (delta-24-rgd) in patients with recurrent glioblastoma	Completed	NCT01582516
12		DNX-2440 (oncolytic AdV)		Recurrent GBM	I	DNX-2440 oncolytic adenovirus for recurrent glioblastoma	Active	NCT03714334
13		DNX-2401 (conditionally replicative and oncolytic AdV)	IFN-γ	Recurrent GBM or gliosarcoma	Ib	DNX-2401 with interferon gamma (IFN-γ) for recurrent glioblastoma or gliosarcoma brain tumors (TARGET-I)	Completed	NCT02197169
14		DNX-2401 (conditionally replicative and oncolytic AdV)	TMZ	Recurrent GBM	I	Virus DNX2401 and temozolomide in recurrent glioblastoma	Completed	NCT01956734
15		AdV-TK	Ganciclovir + Chemotherapy	Recurrent high grade gliomas	II	ADV-TK improves outcome of recurrent high-grade glioma	Completed	NCT00870181
16		DNX-2401 (conditionally replicative and oncolytic AdV)	Pembrolizumab	GBM or gliosarcoma	II	Combination Adenovirus + pembrolizumab to trigger immune virus effects (CAPTIVE)	Active	NCT02798406
17		DNX-2401 (conditionally replicative and oncolytic AdV)		Recurrent high grade gliomas	I	Oncolytic Adenovirus DNX-2401 in treating patients with recurrent high-grade glioma	Recruiting	NCT03896568
18		Ad-RTS-hIL12^*^	Veledimex	GBM or anaplastic oligoastrocytoma	I	A study of Ad-RTS-hIL-12 with veledimex in subjects with glioblastoma or malignant glioma	Active	NCT02026271
19		Neural stem cells loaded with + oncolytic AdV	Radio and Chemotherapy	Malignant glioma	I	Neural stem cell based virotherapy of newly diagnosed malignant glioma	Completed	NCT03072134
20		Ad-RTS-hIL12	Veledimex	Pedriatic brain tumors or diffuse intrinsic pontine glioma	I	A study of Ad-RTS-hIL-12 + Veledimex in pediatric subjects with brain tumors or DIPG	Active	NCT03330197
21	HSV-1	M032-HSV^*^+ IL-12		Recurrent GBM, progressive GBM, anaplastic astrocytoma or gliosarcoma	I	Genetically engineered HSV-1 phase 1 study for the treatment of recurrent malignant glioma (M032-HSV-1)	Recruiting	NCT02062827
22		C134-HSV^*^+ IRS-1		GBM, anaplastic astrocytoma, gliosarcoma	I	Trial of C134 in patients with recurrent GBM (C134-HSV-1)	Recruiting	NCT03657576
23		G207 (oncolytic HSV-1)		Recurrent brain cancer	I-II	Safety and effectiveness study of G207, a tumor-killing virus, in patients with recurrent brain cancer	Completed	NCT00028158
24		G207 (oncolytic HSV-1)	Radiotherapy	Pediatric recurrent or refractory cerebellar brain tumors	I	HSV G207 in children with recurrent or refractory cerebellar brain tumors	Recruiting	NCT03911388
25		G207 (oncolytic HSV-1)	Radiotherapy	Pediatric progressive or recurrent supratentorial brain tumors	I	HSV G207 alone or with a single radiation dose in children with progressive or recurrent supratentorial brain tumors	Recruiting	NCT02457845
26		G47delta (oncolytic HSV-1)	TMZ	GBM	II	Results of a phase II clinical trial of oncolytic herpes virus G47Δ in patients with glioblastoma	Completed	Todo, 2019
27	LV	Temferon^*^		GBM with unmethylated MGMT promoter	I-IIa	A Phase I/IIa study evaluating temferon in patients with glioblastoma and unmethylated MGMT (TEM-GBM)	Recruiting	NCT03866109
28		NK-92/5.28.z^*^		GBM	I	Intracranial injection of NK-92/5.28.z cells in patients with recurrent HER2-positive glioblastoma (CAR2BRAIN)	Recruiting	NCT03383978
29		Modified γδ T cells, resistant to chemotherapy (DRI^*^)	TMZ	GBM	I	Novel gamma-delta (γδ)T cell therapy for treatment of patients with newly diagnosed Glioblastoma (DRI)	Recruiting	NCT04165941
30		CAR T cells with a chlorotoxin tumor targeting domain		Recurrent or progressive MPP2^+^ GBM, recurrent grade III glioma, recurrent grade II glioma	I	Chimeric antigen receptor (CAR) T cells with a chlorotoxin tumor-targeting domain for the treatment of MPP2^+^ recurrent or progressive glioblastoma	Recruiting	NCT04214392
31		IL13Rα2-specific hinge optimized 41BB-co-stimulatory CAR truncated CD19^+^ autologous T cells^*^		Recurrent or refractory GBM	I	Genetically modified T-cells in treating patients with recurrent or refractory malignant glioma	Recruiting	NCT02208362
32		HER2(EQ)BBzeta/CD19t^+^ T cells^*^		Recurrent or refractory GBM	I	Memory-enriched T cells in treating patients with recurrent or refractory grade III-IV glioma	Recruiting	NCT03389230
33		Autologous CD8^+^ T cells that express IL13ζ CAR and HSV-TK	Ganciclovir	Recurrent or refractory high-grade malignant glioma	I	Cellular adoptive immunotherapy using genetically modified T-lymphocytes in treating patients with recurrent or refractory high-grade malignant glioma	Completed	NCT00730613
34	MV	Carcinoembryonic Antigen (CEA)		Recurrent GBM	I	Viral therapy in treating patients with recurrent glioblastoma multiforme	Completed	NCT00390299
35	NDV	NDV-HUJ		Recurrent GBM	I-II	Phase I/II trial of intravenous NDV-HUJ oncolytic virus in recurrent glioblastoma multiforme	Completed	Freeman et al., 2006
36	RV	Autologous T cells expressing receptors anti-mutated neoantigens		GBM, non-small cell lung cancer, ovarian cancer, breast cancer, gastrointestinal cancer, genitourinary cancer	II	Administration of autologous T-cells genetically engineered to express T-cell receptors reactive against mutated neoantigens in people with metastatic cancer	Recruiting	NCT03412877
37		Toca 511	Toca FC	Recurrent GBM, anaplastic astrocytoma, anaplastic oligodendroglioma, anaplastic oligoastrocytoma	I	A study of a retroviral replicating vector combined with a prodrug administered to patients with recurrent malignant glioma	Completed	NCT01156584
38		Leukocytes expressing anti-EGFRvIII CAR^*^	Aldesleukin, Fludarabine, Cyclophosphamide	Malignant glioma, GBM, brain cancer, gliosarcoma	I-II	CAR T cell receptor immunotherapy targeting EGFRvIII for patients with malignant gliomas expressing EGFRvIII	Completed	NCT01454596
39		Autologous HER2-CD28 CMV-T cells		GBM	I	CMV-specific cytotoxic T lymphocytes expressing CAR TARGETING HER2 in patients with GBM (HERT-GBM)	Completed	NCT01109095
4		CD34^+^ cells are transduced with a fibronectin assisted RV expressing MGMT	Filgrastim, iomustine, procarbazine hydrochloride, vincristine sulfate	Bone marrow suppression, brain and CNS tumors	I	Combination chemotherapy plus gene therapy in treating patients with CNS tumors	Completed	NCT00005796
41		Neural stem cells that express cytosine deaminase	5-fluorocytosine	Recurrent high-grade gliomas	Pilot	A pilot feasibility study of oral 5-fluorocytosine and genetically-modified neural stem cells expressing *E. coli* cytosine deaminase for treatment of recurrent high grade gliomas	Completed	NCT01172964
42		Toca 511	Toca FC ± Iomustine, bevacizumab	Recurrent GBM, anaplastic astrocytoma, anaplastic oligodendroglioma, anaplastic oligoastrocytoma	I	Study of a retroviral replicating vector combined with a prodrug to treat patients undergoing surgery for a recurrent malignant brain tumor	Completed	NCT01470794
43		Toca 511	Toca FC	Recurrent GBM, anaplastic astrocytoma, anaplastic oligodendroglioma, anaplastic oligoastrocytoma	I	Study of a retroviral replicating vector given intravenously to patients undergoing surgery for recurrent brain tumor	Completed	NCT01985256
44		Chemoprotected autologous stem cells	Radiation, carmustine, O6-benzylguanine	GBM or gliosarcoma	I-II	O6-benzylguanine-mediated tumor sensitization with chemoprotected autologous stem cell in treating patients with malignant gliomas	Active	NCT00669669
45		Allogenic CD8^+^ T cells expressing IL13-ζ and HSV-TK	Aldesleukin	Recurrent or refractory malignant glioma	I	Phase I study of cellular immunotherapy for recurrent/refractory malignant glioma using intratumoral infusions of GRm13Z40-2, an allogeneic CD8^+^ cytolitic T-cell line genetically modified to express the IL 13-zetakine and HyTK and to be resistant to glucocorticoids, in combination with interleukin-2	Completed	NCT01082926
46		HSV-TK	Ganciclovir and radiotherapy	GBM	III	A phase III clinical evaluation of herpes simplex virus type 1 thymidine kinase and ganciclovir gene therapy as an adjuvant to surgical resection and radiation in adults with previously untreated glioblastoma multiforme	Completed	Rainov, 2000
47	RV vs. AdV	HSV-TK	Ganciclovir	Malignant glioma		Thymidine kinase gene therapy for human malignant glioma, using replication-deficient retroviruses or adenoviruses	Completed	Sandmair et al., 2000
48	VACV	TG6002 (oncolytic VACV) + FCU1^*^	5-FC	Recurrent GBM	I	Safety and efficacy of the oncolytic virus armed for local chemotherapy, TG6002/5-FC in recurrent GBM patients (ONCOVIRAC)	Recruiting	NCT03294486
49	PVS	PVSRIPO^*^		Recurrent GBM	I	PVSRIPO for recurrent GBM	Active	NCT01491893
50	H-1PV	ParvOryx		Progressive primary or recurrent GBM	I-II	Parvovirus H-1 (ParvOrxy) in patients with progressive primary or recurrent GBM. (ParvOryx01)	Completed	NCT01301430

* *(18) IL-12 under the transcriptional control of the RheoSwith Therapeutic System (RTS). (21) M032 is an oncolytic HSV that only infects and kills tumor cells. (22) C134 is an oncolytic HSV that safely replicate and kill glioma cells. (27) Temferon: autologous CD34^+^-enriched hematopoietic stem and progenitor cells exposed to transduction with a lentiviral vector driving myeloid specific interferon-alpha2 expression. (28) The NK-92/5.28.z cell line (also referred to as HER2.taNK) represents a stable, lentiviral-transduced clone of ErbB2 (HER2)-specific, second-generation CAR-expressing derivative of clinically applicable NK-92 cells. 29) DRI, Drug resistant immunotherapy. (31) A preparation of ex vivo expanded, genetically modified autologous central memory-enriched T-cells (Tcm) transduced with a replication-incompetent, self-inactivating (SIN) lentiviral vector expressing a hinge-optimized, chimeric antigen receptor (CAR) specific for interleukin-13 receptor alpha 2 (IL13Ra2), and containing the cluster of differentiation 137 (CD137; 4-1BB) co-stimulatory signaling domain fused to the signaling domain of the T cell antigen receptor complex zeta chain (CD3-ζ), and a truncated form of human cluster of differentiation 19 (CD19t). (32) A preparation of genetically modified autologous central memory (Tcm) enriched T cells transduced with a lentiviral vector expressing a chimeric antigen receptor (CAR) consisting of an anti-human epidermal growth factor 2 (HER2) single chain variable fragment (scFv) derived from trastuzumab, with a 4-1BB (CD137) costimulatory domain that is linked to the signaling domain of the T-cell antigen receptor complex zeta chain (CD3-zeta) (BBz), and truncated CD19 (CD19t). (38) CAR, chimeric antigen receptor. (48) FCU1 encodes a bifunctional fusion protein that converts 5-FC into 5-FU. (49) PVSRIPO is an attenuated chimera that restricts the virus to infect CNS cells but not spinal cord motor neurons*.

### Suicide Gene Therapy: Conditional Cytotoxic Therapy

Suicide gene therapy is the most studied gene therapy approach for the treatment of glioma. This strategy is based on genes encoding for an enzyme that converts a non-toxic prodrug into a cytotoxic drug. Gene therapy vectors allow restricting enzyme expression to the transduced brain tumor cells, without altering the normal brain parenchyma. In addition, this strategy is toxic for cells that are replicating, and thus, specifically targets dividing tumor cells.

Genetically engineered neural or mesenchymal stem cells (NSC, MSC) may be used as vectors for suicide gene therapy, given their ability to migrate toward tumor cells. Recently, Tamura and colleagues evaluated the efficacy of a LV encoding HSV-TK under the control of a tet-inducible system for the treatment of GBM using neural stem/progenitor cells (NS/PCs) derived from induced pluripotent stem cells (hiPSCs). Results showed the directional migration of these NS/PCs and the consequent inhibition in tumor growth in a human GBM xenograft model (Tamura et al., 2020). Currently, there are around 20 Phase-I/II clinical trials testing the effectiveness of AdV in different types of glioma (Table 3). The great majorities of these are studying either the effect of HSV-tk gene therapy or studying the effect of oncolytic AdVs in combination with valacyclovir/ganciclovir or standard of care (SOC) therapies (Chiocca et al., 2011) (Table 3). However, encouraging results from a multi-institutional Phase-II study (NCT00589875) (Wheeler et al., 2016) contrasted with negative results from a Phase-III randomized open-label trial using a similar approach (NCT00870181) (Ji et al., 2016). A Phase-I trial is currently evaluating the intratumoral delivery of Ad-TK and oral administration of the prodrug valacyclovir coupled with SOC and the checkpoint inhibitor Nivolumab in newly diagnosed patients with HGG (NCT03576612). Also, a phase III clinical trial revealed that adjuvant therapy with HSV-tk and ganciclovir through retroviral gene therapy delivered to the surgical resection cavity in combination with radiation in adults with previously untreated GBM failed to improve the overall survival of (Rainov, 2000). Although the feasibility and good biosafety profile of this gene therapy strategy were supported in this study. The failure of this specific protocol may be due mainly to the presumably poor rate of delivery of the HSV-tk gene to the tumor cells. In addition, the current mode of manual injection of vector-producing cells with a non-migratory fibroblast phenotype limits the distribution of these cells and the released replication-deficient RV to the immediate vicinity of the needle track. Further evaluation of the RV-mediated gene therapy strategy must incorporate refinements such as improved delivery of vectors and transgenes to the tumor cells and improved delivery of the prodrug across the BBB and blood-tumor barrier to the transduced tumor cells (Rainov, 2000).

Other clinical trials for recurrent glioblastoma or gliosarcoma evaluated directly injected, genetically modified, conditionally replicative and oncolytic human-derived adenovirus, DNX-2401 in combination with IFNγ (NCT02197169). This trial established an active infection with the virus replicating in, and killing neighboring glioma cells. Similarly, patients with recurrent GBM were also treated with DNX-2401 which was delivered into brain tumor followed by up to two 28-day cycles of oral temozolomide (TMZ) using a schedule of 7 days on/7 days off to evaluate the efficacy of this combination (NCT01956734). Both these clinical trials showed encouraging results with respect to survival outcome. In another Phase-I/II trial (NCT01582516) recurrent GBM patients were treated with replication competent adenovirus i.e., Delta-24-RGD through convection-enhanced delivery (CED), showing similar results. Although these trials offered good safety data and indications of anti-glioma activity, one must await results of Phase 3 clinical trials in order to assess therapeutic benefits.

Another example of a conditional cytotoxic approach involves the expression of the yeast or bacterial enzyme CD in cancer cells, activates the conversion of the prodrug 5-FC into the anticancer drug 5-FU (Takahashi et al., 2014). CD is virtually absent in mammalian cells, which makes 5-FC non-toxic to human cells under normal conditions (Okura et al., 2014). Toca 511, is a replication competent RV encoding CD that has demonstrated to promote tumor eradication in mouse glioma models (Ostertag et al., 2012), together with durable antitumor immune responses (Mitchell et al., 2017). Vocimagene amiretrorepvec (Toca 511) or Toca 511 with flucytosine (Toca FC) have been evaluated in Phase I clinical trials which demonstrated safety and good tolerability, with tumor regression at the site of infusion and durable responses in patients with recurrent high-grade glioma (Cloughesy et al., 2018). However, a recent phase III clinical trial (NCT02414165) revealed that among 403 randomized patients who underwent tumor resection for first or second recurrence of GBM or anaplastic astrocytoma, administration of Toca511 or Toca FC compared with standard of care did not improve overall survival or other efficacy end points (Cloughesy et al., 2020).

Novel conditionally cytotoxic enzymes have been recently developed i.e., a novel isocytosine deaminase (ICD) named Vcz converts the prodrug 5-fluoroisocytosine (5-FIC) into 5-FU (Kazlauskas et al., 2019) and the purine nucleoside phosphorylase (PNP), which converts the prodrug fludarabine phosphate (F-araAMP) to diffusible toxic fludarabine (2-F-araA; 2-FA), these have yet to reach testing in the clinical arena.

#### Targeted Toxins

Toxins have been evaluated in several anti-glioma studies targeting IL13Rα2, the urokinase-type plasminogen activator (uPA) receptor, growth factor receptors and transferrin receptors, due to their differential expression status in glioma cells when compared to normal brain cells (Candolfi et al., 2011; Castro et al., 2011). The natural ligands of these receptors are fused to the catalytic and fusion domains of cytotoxic bacterial products such as *Pseudomonas* and *Diphtheria* exotoxins, which are then internalized and cause apoptosis within glioma cells.

Our group developed a regulatable AdV encoding a mutated human IL-13 fused to *Pseudomonas* exotoxin (PE), under the control of the tet-inducible promoter system that specifically binds to IL13Rα2, expressed by GBM cells that differs from the physiological IL4R/IL13R receptor (Candolfi et al., 2010). When comparing this AdV with the hIL-13-PE protein formulation used in clinical trials (Cintredekin Besudotox) and a second-generation mhIL-13-PE, we found that even though both proteins exhibited severe neurotoxicity Ad-mediated delivery of IL-13-PE, in the presence of Doxycycline, led to tumor regression and long-term survival in over 70% of the animals without apparent neurotoxicity (Candolfi et al., 2010).

### Tumor Suppressor Gene Therapy

The aim of tumor suppressor gene therapy is to restore the function of tumor suppressor genes which are commonly inactivated in glioma cells. These genes can regulate diverse cellular functions including cell-cycle regulation, regulation of cellular proliferation and death and DNA damage repair system.

#### TP53 Gene

p53 is well-documented tumor suppressor gene located on chromosome 17p. Inactivation of p53 is one of the most commonly mutated tumor suppressors in glioma which accounts for ~50% in grade II and III glioma, 25–30% in primary and 60–70% in secondary GBM (England et al., 2013). Tumor suppressor gene therapy using p53 as a target was first tested by delivering through replication-deficient adenovirus (Kwiatkowska et al., 2013). The most commonly used adenoviral vector for p53 is the type 5 adenovirus in which the E1 region is replaced with the cDNA of the wild type p53 gene and is driven under the control of a CMV promoter (Ad5CMV-p53) (Cirielli et al., 1999; Lang et al., 1999; Li et al., 1999; Shono et al., 2002). There was a marked inhibition of growth in implanted gliomas and significant prolongation of survival of animals following the delivery of wild type p53 gene (Badie et al., 1998; Cirielli et al., 1999; Li et al., 1999). Delivery of wild type p53 also suppressed angiogenesis in GBM (Van Meir et al., 1994). SGT-53 is a transferring receptor-targeted liposomal vector encapsulating wild-type p53 plasmid DNA that can cross the BBB and target GBM cells. This resulted in a reduction of MGMT and induction of apoptosis in GBM xenografts mice (Kim et al., 2014). Ad5CMV-p53 can be most effective when used in combination with radiation and chemotherapy (Biroccio et al., 1999; Shono et al., 2002). Similarly, combined treatment of p53 transfection with FasL, GM-CSF, and B7-1 gene enhances apoptosis and inhibits cell growth (Shinoura et al., 2000; Pan et al., 2010). All these results led to phase-I trials of Ad5CMV-p53 gene therapy in recurrent malignant glioma (NCT00004041, NCT00004080). In another study, combination of baculovirus mediated delivery of p53 gene with sodium butyrate, a histone deacetylase inhibitor markedly reduced the growth of glioma cells and enhanced the survival of glioma bearing animals (Guo et al., 2011).

Systemic delivery of a nano-platform encapsulating wild type p53 (scL)-p53 also sensitizes cancer stem cells (CSCs) and bulk tumor cells to TMZ and increase apoptosis (Kim et al., 2014). In another study, Misra et al. generated a p53-EGFP-C3 fusion construct which expressed GFP to allow an estimation of p53 mediated anti-glioma activity and delivered them to glioma cells through a cationic cholesterol based nanocarrier prepared by mixing cationic cholesterol Gemini (ChoL-5L) with natural lipid DOPE in a molar 1:4 ratio. Introduction of wild type p53 cDNA through this nanocarrier induced apoptosis and significantly reduced the tumor volume in mice (Misra et al., 2014). Similarly, a nanoplatform assembled by coupling β-cyclodextrin and the cationic polymer polyethyleneimine to a hydrophobic polymer pullulan (PPEICD) was used to codeliver the antitumor drug mitoxantrone and wild type p53 cDNA to glioma cells. Herein β-cyclodextrin serves as a nanocontainer for mitoxantrone while the cationic part can condense p53 cDNA. Delivery of this nanocomplex induced cell death in glioma cells (Mitha and Rekha, 2014).

#### p16 Gene

P16/CDK4/Rb/E2F is the most commonly altered pathway in gliomas. Therefore, over-expression of p16 gene through recombinant replication-deficient adenovirus significantly reduced the invasion of glioma by suppressing the activity of MMP2 (Chintala et al., 1997). Moreover, data from a previous study revealed that retroviral delivery of p16^INK4A^ gene could effectively inhibit the progression of glioma but only when endogenous pRb is intact (Xande et al., 2020). Similarly, intratumor injection of pCL retrovirus encoding full-length human p16 cDNA resulted in 95% reduction of gliomas *in situ* through necrosis and cell-cycle arrest (Hung et al., 2000). Another study also revealed that adenoviral delivery of p16 gene enhanced radiation induced cell killing possibly by a non-apoptotic mechanism with abnormal nucleation in glioma cells (Hama et al., 2003). Moreover, restoration of the wt-p16 activity into p16-null SNB19 glioma cells significantly inhibited tumor cell invasion (Chintala et al., 1997). Similarly, down-regulation of integrin α(v)β(3) expression and integrin-mediated signaling in glioma cells by adenoviral transfer of antisense urokinase-type plasminogen activator receptor and wild type p16 cDNA resulted in decrease adhesion, migration, proliferation and enhanced survival (Adachi et al., 2001, 2002). However, sometimes cell-cycle arrest following transfer of p16 gene to glioma cells resulted in the development of chemoresistance to some cytotoxic drugs such as cisplatin, paclitaxel, topotecan and ACNU (Fueyo et al., 1998; Hama et al., 1998).

Deregulation of E2F transcription factor, specifically E2F-1 is a critical target of any alteration of the p16/Rb/E2F pathway in glioma. E2F-1 positively regulates the transcription of S-phase genes and drives the cell-cycle progression through G1 checkpoint. Study revealed that transfer of E2F-1 along with p53 to gliomas induced apoptosis and appeared to be more effective than wild type p53 as it can induce apoptosis even in p53 resistant glioma cells (Fueyo et al., 1998). In fact, vectors expressing p16 and p21 were more effective than wild type p53 at improving survival (Wang et al., 2001). Thus, Adenovirus mediated transfer of E2F-1 alone or in combination with wild type p53 to glioma cells should propel the development of clinical trials for glioma treatment. Deregulated p16 expression also plays a crucial role in angiogenesis in glioma. Therefore, transfer of p16 cDNA through recombinant replication-defective adenoviral vector to glioma cells markedly inhibited angiogenesis through suppressing vascular endothelial growth factor (VEGF) expression (Harada et al., 1999).

However, in a recent report, when p16^INK4A^ was expressed under the control of Tet repressor system in glioma cells on a long-term basis, it decreased the expression of Rb, suggesting that this gene therapy approach involving p16^INK4A^, could ultimately have led to the selection of Rb-deficient gliomas (Simon et al., 2002).

#### PTEN Gene

Phosphatase and Tensin Homolog on chromosome number 10 (PTEN) is a tumor suppressor gene which contains a central catalytic phosphatase core domain that negatively regulates PI3K by dephosphorylation of PIP_3_ to PIP_2_ and can act as an excellent target for gene therapy (Kanu et al., 2009). PTEN is inactivated in 33% of all gliomas resulting in aberrant activation of PI3K pathways (Dunn et al., 2012). Therefore, the transfer of chromosome 10 to glioma cells induced thrombospandin-1 and inhibited angiogenesis in glioma (Hsu et al., 1996). Restoration of PTEN activity in glioma cells led to suppression of their neoplastic phenotype (Cheney et al., 1998). Forced PTEN expression through AdV conferred sensitivity to temozolomide and/or ionizing radiation (Inaba et al., 2011). Adenoviral re-expression of PTEN in glioma cells inhibited Akt kinase activity, leading to tumor cell apoptosis (Davies et al., 1998). Additionally, adenoviral expression of PTEN demonstrated an anti-angiogenic response in glioma along with decreased proliferation and increased apoptosis in gliomas *in vivo* (Abe et al., 2003; Lu et al., 2004). Another study revealed that replication-defective adenoviral vector, i.e., MMCB mediated PTEN gene transfer to malignant glioma inhibited the growth and survival of the tumor cells, suppressing the tumorigenecity of malignant gliomas (Cheney et al., 1998). It has been found that, overexpression of EGFR and mutation/deletion of PTEN is one of the main genetic changes identified in gliomas. It was demonstrated that combined infection of glioma cells with antisense-hTERT and wt-PTEN bearing adenovirus significantly inhibited proliferation and reduced tumor load both *in vivo* and *in vitro* (You et al., 2007). Similarly, introduction of an expression plasmid carrying shRNA against hEGFR and wt-PTEN cDNA to glioma cells significantly suppressed the tumor cell proliferation, reduced the tumor invasion and promoted tumor cell apoptosis in gliomas (Han et al., 2010). TIMPs (the inhibitors of MMP2) and PTEN are known to be inhibitors of the invasive activities of malignant gliomas. Therefore, adenoviral delivery of TIMP2 and PTEN/MMAC1 cDNA to human glioma cells significantly inhibited invasive phenotype and growth of gliomas *in vivo* (Lu et al., 2004).

### Gene Therapy Targeting Signaling Pathways

#### EGFR and EGFRvIII

EGFRvIII is the most common variant, leading to constitutively active EGFR signaling in glioma (Gan et al., 2013). EGFRvIII is often co-expressed with full-length EGFR in glioma cells. This complicates our understanding of its contribution to tumorigenesis (Shinojima et al., 2003; Fan et al., 2013). Delivery of both viral and non-viral vectors containing antisense-RNA to target EGFRvIII into intracranial glioma xenografts reduced tumor load significantly (Shir and Levitzki, 2002). Treatment with antisense-RNA or siRNA of U251 glioma expressing EGFRvIII also reduced tumor volume (Kang et al., 2006). This EGFR specific siRNA is directed against the TK-domain and were shown to cause 90% knockdown of EGFR mRNA (Kang et al., 2006). Thus, the overall median survival increased by almost 90% (Kang et al., 2006). Blocking the gene expression of both EGFR and β-catenin significantly inhibited the glioma invasive ability (Wang et al., 2013). It was shown that cyclodextrin-modified dendritic polyamine complexes (DexAMs) were effective at delivering EGFRvIII siRNA efficiently and selectively to glioblastoma with minimal toxicity (Kim et al., 2011). Furthermore, co-delivery of EGFRvIII siRNA and erlotinib in GBM was found to significantly inhibit cell proliferation and induce apoptosis in glioblastoma cells (Kim et al., 2011). Similarly, the use of an expression plasmid Pgenesil-1 vector viz. psiRNA-EGFR-PTEN on U251 glioma resulted in the suppression of cell proliferation, arrest of cell cycle, reduction of cell invasion and promotion of apoptosis both *in vitro* and *in vivo* (Han et al., 2010). Herein the vector expresses a small hairpin RNA-targeting EGFR and wild-type PTEN cDNA in glioma cells (Han et al., 2010). In addition, ribozyme targeting EGFRvIII inhibits ERM5-1 and U87MG GBM cells (Halatsch et al., 2000). Herein, anti-EGFRvIII hairpin ribozyme resulted in significant reduction in glioma proliferation (Halatsch et al., 2000). Moreover, treatment with anti-EGFRvIII hairpin ribozymes was shown to reduce EGFRvIII mRNA by 90% and inhibit anchorage-independent growth of U87MG glioma cells (Karpel-Massler et al., 2009). On the other hand, adjuvant miRNA-based therapies also showed potential for glioma treatment. miR-7 appears to be an effective inhibitor of the EGFR signaling in glioma by direct inhibition of the EGFR and down-regulation of Akt signaling, leading to decreased invasiveness of glioma. miR-7 treatment also helped to overcome the radio-resistance properties of glioma (Padfield et al., 2015). Taking into account both the preclinical and clinical experience of targeting the EGFR signaling pathway for GBM therapeutics, it can be concluded that as a monotherapy this approach is unlikely that it will work in the clinical arena, due in part to the heterogeneity of GBM and also the numerous alternative growth promoting pathways that are used by glioma cells. Nevertheless, targeting the EGFR pathway would be a valuable adjuvant strategy to be used in combination with other therapeutic approaches.

#### VEGF

The expression of VEGF is up-regulated in gliomas. Therefore, targeting VEGF could a promising approach for glioma management. It was shown that efficient delivery of anti-sense VEGF cDNA *via* an adenoviral Ad5CMV-αVEGF vector, into subcutaneous human glioma tumors established in nude mice, inhibited tumor growth (Im et al., 1999). Moreover, direct intra-tumoral injection of a VEGF siRNA-encoding plasmid complexed with linear PEI, efficiently reduced the vascularization of tumors in xenografts (Niola et al., 2006). Like VEGF, high-affinity VEGF receptor Flk-1/KDR (VEGFR-2) also plays a key role in tumor angiogenesis. Strategies to block VEGFR-2 signaling were successfully used to inhibit experimental tumor growth as this is the main signaling axis required for the proliferating tumor endothelium. It has been found that retroviral delivery of mutant-VEGFR1 that lacks the intracellular tyrosine kinase domain led to a strong reduction of glioma growth and angiogenesis in a xenografted C6 glioma model (Heidenreich et al., 2004). Also, the retroviral transfer of full-length VEGFR-1 cDNA caused a significant reduction of glioma growth. The inhibitory effects of the VEGFR-1 mutants and the full length VEGFR-1 were mediated through host tumor endothelial cells. The formation of heterodimers between VEGFR-2 and full length or truncated VEGFR-1 might contribute to the glioma inhibitory effect by modulating distinct signal transduction pathways (Heidenreich et al., 2004). Soluble vascular endothelial growth factor receptor (sFlt-1) also plays an important role in anti-glioma treatment. Co-delivery of sFlt-1 and angiostatin-endostatin fusion gene (Statin-AE) through non-viral sleeping-beauty (SB) transposons to glioma xenografts showed marked reduction in tumor vessel density and tumor load (Ohlfest et al., 2005). Similarly, co-infection of glioma cells with both anti-angiogenic gene therapy vectors Ad-Flk1-Fc, which expresses a soluble VEGF receptor and oncolytic virus dl922/947 whose replication and subsequent cytotoxicity are restricted to cancer cells, yielded significantly higher anti-glioma effect than monotherapy (Thorne et al., 2006). In another study, construction of an oncolytic adenovirus-based shRNA expression system i.e., Ad-DeltaB7-shVEGF revealed a marked reduction in glioma vasculature and tumor load *in vivo*. This study also demonstrated that the duration and magnitude of VEGF silencing by Ad-DeltaB7-shVEGF was greater than the efficacy elicited by the replication-incompetent adenovirus expressing sh-VEGF (Ad-DeltaE1-shVEGF) (Yoo et al., 2007). The delivery of a replication-incompetent adenovirus expressing, VEGF promoter-targeted transcriptional repressor Cys2-His2 zinc-finger proteins, F435-KOX namely Ad-DeltaE1-KOX significantly reduced angiogenesis and tumor load (Kang et al., 2008). Likewise, using the previously mentioned oncolytic adenovirus Ad-DeltaB7 expressing F435-KOX, namely Ad-DeltaB7-KOX, elicited similar anti-glioma efficacy in a human xenograft model (Kang et al., 2008). VEGF and high-affinity VEGF receptor Flk1/KDR (VEGFR2) are key regulators of glioma angiogenesis, thus, inhibition of VEGFR2 expression would inhibit the development of new blood vessels within the tumor microenvironment (TME) and inhibit glioma progression. Data also revealed that delivery of genetic sequences of antisense RNAs to alter the splicing pattern and expression of the VEGFR2 transcript using pAAV-U7-smOPT vector markedly reduced glioma growth *in vivo* (Muralidharan et al., 2019).

### Blood Brain Barrier Disruptive Gene Therapy

Treatment of gliomas could be improved markedly by the development of non-invasive therapeutic approaches that elicit robust, endothelial cell-selective gene expression in specific brain regions. Focused ultrasound (FUS) is one such targeted and non-invasive technique that can be used to activate gas filled microbubbles (MBs) to oscillate within the bloodstream. MBs expand and contract upon sonication by FUS producing cavitation. Stable cavitation is induced by relatively lower amplitude of FUS zzzzz. Generally, FUS elicits endothelial selective transfection without opening the BBB. Study found that magnetic resonance (MR)-guided MB enhanced low intensity pulsed FUS (LIFU) transiently open the BBB and delivers a liposome loaded MGMT inhibitor, O^6^-(4-bromothenyl) guanine (O^6^BTG) in mice bearing TMZ-resistant gliomas, thereby sensitizing murine and human gliomas to TMZ both *in vivo* and *in vitro* (Papachristodoulou et al., 2019). In another study, researchers developed a VEGFR2-targeted and cationic microbubble (VCMB) gene vector with FUS exposure to allow transient gene delivery. They delivered pHSV-TK/GCV with VCMB under FUS exposure for transgene expression and antitumor effect (Chang et al., 2017). It was also found that there was a significant increase in median survival following single treatment of FUS with doxorubicin in 9L gliosarcoma bearing rats (Treat et al., 2012). Another example is 1,3-bis(2-chloroethyl)-1-nitrosourea (BCNU) which showed only a relatively limited effect against glioma. However, FUS-mediated delivery of BCNU to glioma-bearing rats greatly increased the intracellular retention and inhibition of tumor progression *in vivo* (Deng et al., 2019). Moreover, Fan et al. fabricated PEG-b-PMBSH-loaded MBs which are formed by boron-containing nanoparticles coupling with MBs for the treatment of GL-261 bearing mouse glioma model (Fan et al., 2019). Thus, FUS in conjunction with MBs has emerged as a unique non-invasive modality for MR image-guided gene delivery to the brain which involves transient disruption of BBB which may induce a sterile inflammatory response. It was found that activating circulating cationic plasmid bearing MBs with pulsed low pressure (i.e., 0.1 MPa) 1.1-MHz FUS facilitates sonoselective gene delivery to the endothelium selectivity varied inversely with the FUS pressure that means with high pressures i.e., 0.3 MPa and 0.4 MPa FUS consistently inducing BBB opening and extravascular transfection.

## Immune Stimulatory Gene Therapy

### Cytokine Mediated Gene Therapy

Cytokine mediated gene therapy involves tumor-selective gene transfer and *in situ* expression of various cytokine genes such as IL2, IL4, IL12, and IFNβ/γ which can induce robust immune responses to glioma cells (Iwami et al., 2010; Tobias et al., 2013). Gliomas can effectively evade the host immune response (Natsume and Yoshida, 2008; Kwiatkowska et al., 2013). The unique characteristics of the CNS immune system in the context of an intracranial glioma, these include a paucity of antigen-presenting DCs, high levels of anti-inflammatory TGF-β and expression of immune checkpoint molecules by glioma cells and tumor infiltrating immunosuppressive cells. These mechanisms play important roles to protect the CNS from immunological attack. Therefore, it is challenging to stimulate the system to develop an effective anti-glioma response (Assi et al., 2012). The susceptibility of glioma stem cells to the cytotoxic effects of the immune system provides the basis for development of anti-glioma immune gene therapy.

#### Interferon β/γ

IFNβ is a pleiotropic cytokine with antitumoral activity. Therefore, when h-IFNβ expressing adenoviral vector viz. Ad.hIFNβ was introduced into human gliomas stereotactically, it induced increase amount of tumor cell apoptosis *in vivo* (Chiocca et al., 2008). Local administration of intracranial IFNβ gene delivery through adeno-associated viral vectors viz. AAV/P2-Int-mIFNβ also successfully treats orthotopic gliomas with concomitant activation of microglia surrounding the tumors. It is interesting to note that treatment with TMZ prior to AAV-IFNβ abrogated any benefits from the later, while the reverse order of treatment doubled the median survival compared to control population (GuhaSarkar et al., 2017). Moreover, cationic liposome mediated IFNβ gene transfer significantly changes antitumor immune responses and inhibits neovascularization. Many gliomas showed necrotic changes and increased infiltration of CD8^+^ T-cells and macrophages within the tumor following administration of Ad.hIFNβ (Wakabayashi et al., 2008). A phase I/early phase II clinical trial demonstrated the safety and efficacy of this liposomal approach to deliver a plasmid coding for IFN-β in patients with recurrent malignant gliomas following resection of the tumor (Yoshida et al., 2004). This study revealed that there is upregulation of transgene expression and antitumor activity in most of the patients recruited for the study.

Direct injection of the IFNβ gene with a replication deficient adenovirus demonstrated tumor regression in human glioma xenograft, through the activation of NK cells. It also enhanced the generation of DC, T_H_ and macrophage cells and stimulated the generation of cytotoxic T-cells activity. Survival was significantly increased in glioma bearing mice (Qin et al., 2001).

Similarly another proinflammatory cytokine IFNγ, produced by NK, DC, and T-cells diminishes the invasive phenotype of glioma cells by inhibiting its interactions with extracellular matrix molecules (Schroder et al., 2004). Use of adenovirus expressing TNFα or IFNγ into tumors enhanced infiltration of CD4^+^ and CD8^+^ T cells along with increased expression of MHCI/II on the glioma cells *in vivo*. Intracranial administration of both these genes significantly increases the survival of glioma bearing animals (Ehtesham et al., 2002). In the ongoing trials, CD34^+^-enriched hematopoietic stem and progenitor cells (HSPCs), NK-cells or different CAR T-cells are administered either with TMZ or with ganciclovir (Table 3). For instance, in a Phase-II trial, patients with GBM who have an unmethylated MGMT promoter administered with single dose of autologous CD34^+^-enriched HSPCs exposed to transduction with a 3rd generation LV driving myeloid-specific IFN-α2 expression (NCT03866109) (Table 3).

#### IL12

IL12 is one of the most potent anti-tumor cytokines, driving a Th1 response in tumor bearing animals (Tatsumi et al., 2003). Despite its therapeutic success in multiple animal models of cancer, the utility of systemically administered recombinant cytokine has been limited by its toxicity. This has encouraged the development of local IL12 delivery systems through gene transfer. Mice bearing GL-26 gliomas in the right corpus striatum when treated with direct intratumoral administration of replication-deficient adenoviral AdmIL-12 vector, it significantly prolonged the survival of glioma bearing animals with robust infiltration of CD4^+^ and CD8^+^ T-cells (Liu et al., 2002). Another study using vaccinia virus expressing IL12 resulted in effective inhibition of subcutaneous C6 glioma growth in mice (Chen et al., 2001). Moreover, combination therapy of glioma with recombinant vaccinia virus mediated IL2 expression, resulted in significant tumor inhibition with concomitant elevation of NK, Mac-1^+^ and NKT cells in blood and IFNγ and TNFα expression in tumors (Chen et al., 2000, 2001). Neural stem cells (NSCs) isolated from hippocampi of human embryo were used for lipofectamine-mediated transfer of the IL12 gene to rat glioma cells (Yang et al., 2004). Several other groups have delivered IL12 using different non-adenoviral gene therapy vectors. Among them, γ34.5-deleted HSV-1 (oHSV) expressing mouse-IL12 was shown to exert its oncolytic activity and perform better than other IL12 bearing oHSVs in rodent models of GBM (Hellums et al., 2005). Similarly, Semliki forest virus (SVF) vectors were also used for the delivery of hIL-12 gene to RG2 rat glioma model (Roche et al., 2010). SVF carrying IL12 gene alone when administered through an implanted cannula to the brain, reduced the tumor load and prolonged the survival of RG2 glioma bearing animals not only through the oncolytic activity of SVF but also through activating an anti-tumor immune response (Roche et al., 2010). Despite this, the broad tropism of the SVF-based expression vector may limit its use as a glioma gene therapy vector unless this limitation can be overcome. Human umbilical cord blood-derived mesenchymal stem cell (UCB-MSC) have also been used as gene delivery vehicles, i.e., UCB-MSC-IL12M expressing IL12. It was shown that they inhibited GL26 intracranial tumor growth and prolonged survival when administered in the contralateral brain hemisphere (Ryu et al., 2011). Moreover, surviving mice generated memory response against tumor antigens (Ryu et al., 2011). Non-replicative AAV and replicative HSV have also been used to express IL12 in malignant glioma, resulting in significant inhibition of tumor growth and increased expression of IFNγ with microglial activation and recruitment of T and NK cells (Ahn et al., 2016; Barrett et al., 2018). These data demonstrated that cytokine gene therapy through viral vector mediated IL-12 gene expression may be a promising strategy for glioma treatment. Recently, two different Phase-1 dose-escalation trials (NCT02026271, NCT03330197) revealed that when the resection cavity walls were injected with a fixed dose of a regulatable ADV-hIL12 vector i.e., Ad-RTS (RheoSwitch Therapeutic System)-hIL12, together with an oral activator of IL12 expression, veledimex (VDX), the expression of IFNγ increased in peripheral blood in the enrolled patients. To minimize systemic toxicity, the ligand-inducible expression switch, RTS was developed to locally control the production of IL12 in the tumor microenvironment, during fixed periods of time. Also increased infiltration of PD-1^+^ immune population was observed, following Ad-RTS-hIL12 therapy in some of the re-resected tumor samples. Since this was a Phase I trial, it was not powered to assess therapeutic efficacy (Chiocca et al., 2019). Administration of Ad-RTS-hIL12 to glioma patients also revealed pseudo-progression with increased frequencies of tumor infiltrating lymphocytes (TILs) producing IFNγ and expressing PD1 (Chiocca et al., 2019). These inflammatory infiltrates also support an immunological anti-glioma effect of h-IL12 (Barrett et al., 2018; Chiocca et al., 2019).

## Oncolytic Virotherapy

Oncolytic virotherapy (OV) is based on genetically engineered viruses with the ability to infect and replicate within tumor cells and then lyse them, releasing new infectious viral particles that can infect neighboring cells leading to immunogenic cells death and immune stimulation (Figure 1). As such this approach cannot be considered as gene therapy, nevertheless, OV have been engineered to also harbor therapeutic transgenes, which we will discuss briefly below. In this case, they can be considered gene therapeutic platforms. In addition, OVs have been genetically engineered to express therapeutic transgenes to further boost antitumor immunity.

Among all the studied viruses, only one wild-type virus, an oncolytic double-stranded human RNA *orthoreovirus* (referred as reovirus) is under clinical trial as Reolysin in GBM patients (NCT00528684) (Samson et al., 2018). Reovirus is pathologically benign and it is tumor cytotoxic, making it an appealing OV for therapeutic development. Reovirus selectively targets transformed cells with activated Ras signaling pathways and can lyse cancer cells (Zhao et al., 2016). A dose escalation Phase-I clinical trial is currently evaluating the combination of intravenously administered Reolysin and subcutaneously administered GM-CSF in patients with recurrent HGG (NCT02444546).

The first attenuated mutant HSV serotype 1 TK deficient virus, called *dlsptk*, was incapable of replicating in non-dividing cells like neurons but could replicate in human brain tumor cells and kill them *in vitro* (Martuza et al., 1991). HSV1719 is a *first-generation* virus that is devoid of the γ34.5 (Δγ34.5) gene that suppresses PKR/eIF-2a signaling pathway and IFN-induced anti-viral mechanisms. This virus was evaluated in three successful Phase I trials in GBM patients (summarized in Ning and Wakimoto, 2014). The *second*-*generation* vector G207 also contains a gene-disrupting insertion of *lacZ* reporter sequence into U_L_39, a gene encoding for the large subunit of the viral ribonucleotide reductase (ICP6), that is required for replication in non-cycling cells (Aghi et al., 2008). Oncolytic selectivity is thought to occur because mutations in viral ICP6 and γ34.5 functions are respectively complemented by mammalian ribonucleotide reductase and GADD34, whose genes are expressed in cycling cells. Therefore, effective replication of OVs might be limited to a subpopulation of tumor cells, as the majority of tumor cells would not be cycling. This approach provides evidence that ICP6-negative OVs can replicate in quiescent tumor cells carrying specific oncogene deletions, independent of cell-cycle status. G207 successfully completed three trials in the USA, showing a well-tolerated antitumor response when the virus was inoculated after or before the tumor resection (Markert et al., 2000, 2009). Currently, there are two Phase I trials recruiting pediatric patients with recurrent or refractory cerebellar brain tumor (NCT03911388) or supratentioral brain tumors (NCT02457845) to determine the safety of G207 alone or combined with radiotherapy. On the other hand, the vector C134 is a chimeric hCMV/oHSV-1 which encodes the protein kinase R evasion gene IRS1 under the control of human CMV, which maintains the late viral proteins synthesis in malignant glioma cells improving amplification and prolonging survival in two different mouse models implanted intracranially with U87MG and U251MG glioma cells (Shah et al., 2007). A Phase I trial is recruiting GBM patients to evaluate this vector (NCT03657576) (Table 3). The interim analysis of a study using the genetically engineered oncolytic HSV, G47Δ showed that the 1-year survival rate of 13 patients was 92.3% which was significantly higher when compared to 15% survival rate in control population. A study also showed efficient induction of antitumor immunity and successful targeting of cancer stem cells. Another Phase-II trial (NCT00028158) with conditionally replicating oncolytic-HSV1 viz. G207 demonstrated anti-tumor activity and long-term presence of viral DNA in patients, without any serious adverse effects. No patients developed HSV-encephalitis (Markert et al., 2000). Other clinical trials with HSV are still recruiting (Table 3). Moreover, in a Phase-I/II trial, patients with recurrent-GBM were repeatedly administered with oncolytic HUJ, an attenuated lentogenic (nonvirulent) isolate of NDV revealed good tolerability with minimum adverse effects. This finding warrant continued evaluation of NDV-HUJ in GBM (Freeman et al., 2006).

The replication-competent adenovirus DNX-240, marketed as Tasadenoturev, was generated to restrict the viral replication to cells with retinoblastoma pathway deficiency (Fueyo et al., 2003). DNX-240 was first studied in a double-arm Phase-I trial to treat patients with recurrent glioblastoma (rGBM), reporting 20% of patients surviving more than 3 years and three complete responders (NCT00805376) (Lang et al., 2018). Another strategy involves the delivery of neural stem cells transduced with OV Ad5-DNX-2041 or NSC-CRAd-Survivin-pk7 in patients with rGBM and newly diagnosed malignant gliomas respectively (NCT03896568, NCT03072134). Moreover, a Phase II trial is still active, involving the delivery of genetically modified oncolytic adenovirus (DNX-2401) followed by intravenous immune checkpoint inhibitor pembrolizumab to evaluate the treatment efficacy (NCT02798406) (Table 3).

Several studies have shown the therapeutic potential of live attenuated oncolytic polio/rhinovirus recombinant (PVSPIRO) in patients with grade IV malignant glioma to evaluate the efficacy of this vector (NCT02986178) (Table 3). PVSPIRO has tropism toward CD155 that highly expressed in tumor cells, enables infected tumor cell cytotoxicity and stimulation of an inflammatory response (Brown et al., 2017). Finally, a third PVSRIPO-based therapy is ongoing for pediatric patients with rGBM (NCT03043391) (Table 3). Collectively, the successful accrual of these trials will demonstrate whether improved safety, tumor specificity, and efficacy of OVs alone or in combination with other therapies can be translated into the clinic arena.

## Combination Therapies

In an effort to overcome the shortcomings of monotherapies, combination therapies have been developed. Adenoviruses expressing a secretable angiostatin-like molecule (AdK3) in combination with 7.5 Gy radiation dosage in rat C6 gliomas appeared to be more cytotoxic than either treatment alone (Griscelli et al., 2000). Similarly, IL24 can also induce tumor cell death through various mechanisms including endoplasmic stress induced apoptosis, autophagy, anti-angiogenesis and immune activation (Emdad et al., 2009). In GBM models, the anti-tumor effects of Ad-bearing IL24 were also enhanced by radiation (Yacoub et al., 2003a,b). Histone deacetylase (HDAC) inhibitor was also shown to increase Ad-MDA-7/IL24 lethality through ER stress and activation of the extrinsic apoptotic pathways (Dent et al., 2010). Recently, a complex liposome was engineered to carry both a therapeutic gene TRAIL and a cytotoxic drug paclitaxel combined with re-targeting by inserting a peptide angiopep2 that facilitates BBB crossing. This preparation can effectively deliver TRAIL to glioma cells *in vitro* (Sun et al., 2012). Thus, these approaches constitute a valuable adjuvant therapeutic strategies for glioma. Moreover, combination of drugs with different phase specific cytotoxicities such as combination of p19 and p53 gene therapy, where p19 is important to inactivate p53 inhibitors and p53 itself triggers apoptosis, appear promising to target gliomas. Combination therapy with systemically administered liposomal p53 i.e., SGT-53 and TMZ enhanced antitumor efficacy compared to TMZ alone, demonstrating the ability of SGT-53 to improve chemo-sensitivity (Kim et al., 2014, 2015b).

**Figure 2:**
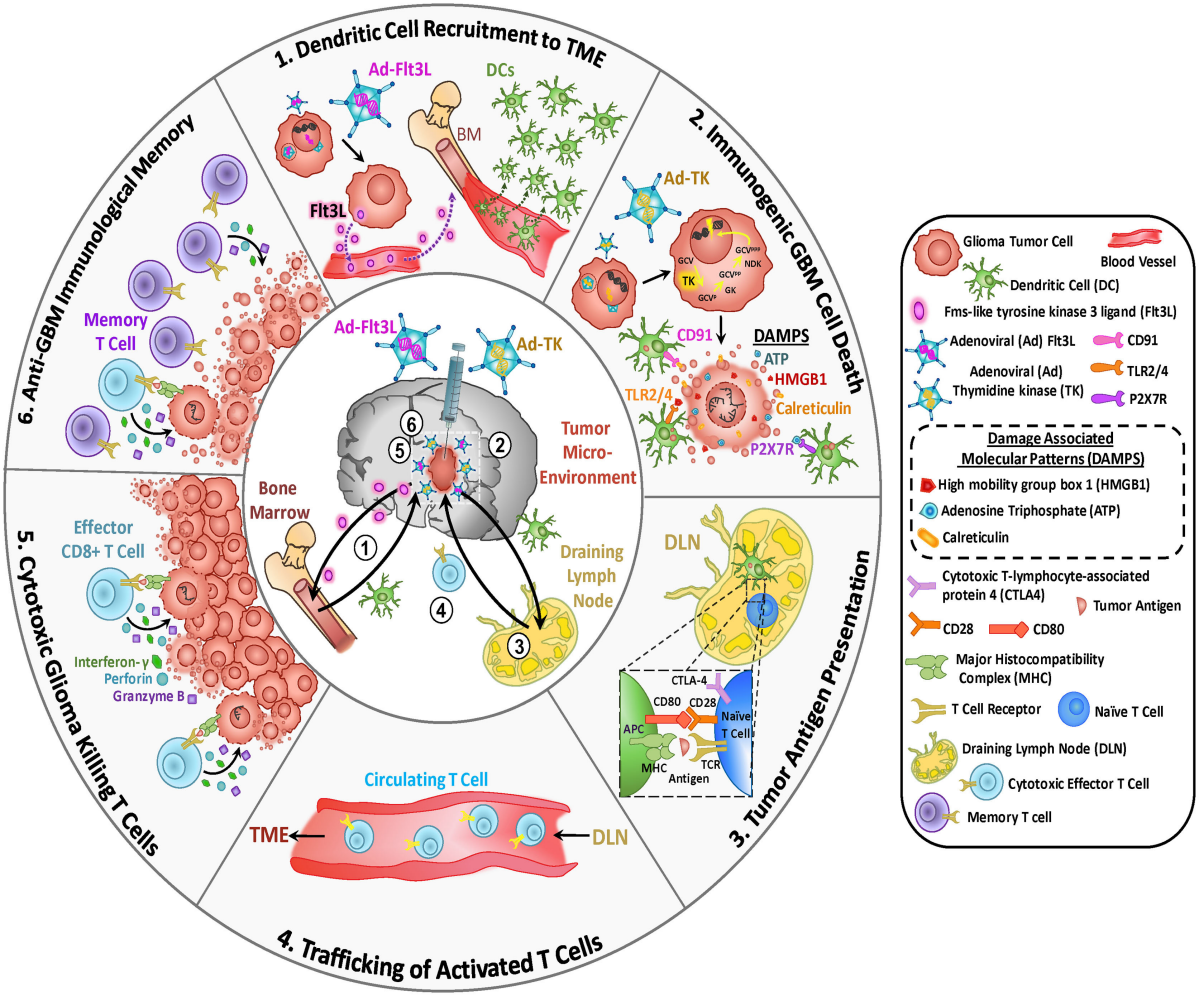
Mechanism underlying the anti-glioma immune response following TK/Flt3L gene therapy. First generation adenoviral vectors (Ad) encoding HSV1-Thymidine Kinase (TK) and HSV1- FMS-like tyrosine kinase 3 ligand (Flt3L) are injected into the tumor cavity following surgical resection. (1) Dendritic Cell Recruitment to Tumor Microenvironment (TME): Tumor cells infected with Ad-Flt3L express Flt3L (pink circles) releasing it into the circulation. Flt3L in the bone marrow (BM) to induces dendritic cells (DCs) expansion, migration, and accumulation within the TME. (2) Immunogenic Glioblastoma (GBM) Cell Death: The prodrug ganciclovir (GCV) is administered systemically. Tumor cells infected with Ad-TK express TK protein which is capable of converting GCV to GCV-monophosphate (GCVp). This intermediate is further phosphorylated by cellular kinases: guanylate kinase (GK) and nucleoside diphosphokinase (NDK). GCV triphosphate (GCVppp) is a purine analog that selectively inhibits DNA replication in proliferating tumor cells leading to DNA breaks and apoptosis. The expression of TK in the presence of GCV mediates the release of damage associated molecular patterns (DAMPs), i.e., HMBG1, Calreticulin, and ATP from dying tumor cells. Expression of Flt3L recruits DCs into the tumor milieu where they take up brain tumor antigens released from the dying glioma cells. These DAMPs bind their corresponding receptors expressed on DCs. HMGB1 binds to TLR2/4, which promotes the production of cytokines and tumor antigen cross-presentation. The binding of extracellular ATP to purigenic receptor P2X7R further promotes the recruitment of DCs. Calreticulin binds to the CD91 receptor, which plays a major role in immunosurvillence. (3) Tumor Antigen Presentation: The DCs loaded with tumor antigens migrate to the cervical draining lymph node (DLN) where they present tumor antigens (Ag) to naïve T cells on MHC, priming tumor specific anti-glioma effector T cells. (4) Trafficking of Activated T cells: Primed CD8^+^ effector T cells enter circulation from DLN and migrate toward the TME. (5) Cytotoxic Glioma Killing T Cells: The tumor specific effector T cells enter the TME and kill residual glioma cells *via* the production of granzyme B, perforin and effector cytokine IFN-y. (6) Anti-GBM Immunological Memory: Continual exposure of T cells to tumor antigens promotes immunological memory. Memory T cells (CD103 and CD69) facilitate an anti-tumor response resulting in inhibition of tumor recurrence.

We have pioneered the combination of Ad-Flt3L and Ad-TK. Combining both these two genes results in GCV phosphorylation which ultimately resulting in tumor cell death (Castro et al., 2014; Kamran et al., 2018a). This induces the release of tumor antigens into the tumor microenvironment and damage-associated molecular pattern molecules (DAMPs), which are molecules that when released into the TME or translocated to the cell membrane during cell death, they trigger an immune response against self-antigens (Kamran et al., 2018a; Altshuler et al., 2020). Our results indicate that release of DAMPs such as HMGB1 from Ad-TK infected tumors is required for the efficacy of Ad-TK+Ad-Flt3L mediated immunotherapy (Candolfi et al., 2009; Curtin et al., 2009). Flt3L increases the migration and infiltration of DCs into the TME. This glioma infiltrating DCs are able to phagocytose antigens that are released during TK-induced glioma cell death (Figure 2) (Curtin et al., 2009; Candolfi et al., 2012). Moreover, HMGB1 activates DCs through TLR2 and then activated DCs transport the antigens to the draining lymph node, generating T-cell mediated cytotoxic immune response (Curtin et al., 2009). This combination therapy provides long-term survival and immunological memory in multiple glioma models. In addition, we have also combined Ad-mediated gene therapy with DC vaccination (Mineharu et al., 2011). We found that compared to either therapy alone, combination of intra-tumoral Ad-Flt3L/Ad-TK with DC vaccination resulted in long-term survival in 90% of glioma bearing animals. Our findings indicated that Ad-Flt3L/Ad-TK modifies the TME that enhances the efficacy of DC vaccination (Figure 2) (Mineharu et al., 2011). Work from our team has also recently shown the combining Ad-Flt3L/Ad-TK-mediated gene therapy together with immune-check point blockade, using CTLA4 or anti-PDL1, it significantly increased median survival when compared with either treatment used independently (Kamran et al., 2017). Similar results were obtained when we tested Flt3L/Ad-TK-mediated gene therapy in combination with depletion of immunosuppressive MDSCs (Kamran et al., 2017). Again, indicating that combination therapies are an attractive way forward to develop novel treatment for GBM.

In our first human Phase-I dose escalation trial (NCT01811992) using a combination of two adenoviral vectors expressing HSV1-tk and Flt3L for the treatment of newly diagnosed, resectable malignant gliomas we observed evidence of biological activity as evidenced by increased frequencies of DCs, CD4 and CD8 T cells within the TME (Lowenstein et al., 2019). Our results showed for the first time that reprogramming of the host's brain immune system to recognize gliomas could present an attractive approach for the treatment of malignant brain tumors (Lowenstein and Castro, 2018).

## Discussion and Conclusions

Although innovative gene-mediated therapies and oncolytic virotherapies (OV) have been developed to treat gliomas, to date, they have failed in improving patients' outcomes compared to current standard of care treatment modalities, including surgery, radiotherapy and chemotherapy. Moreover, drug design and clinical trial implementation all come at a considerable economic cost that often limits the timely development of potentially promising treatments. Opportunities to address the lack of clinical benefit of genetic-based therapies and OV in the clinical arena may be provided by accurate preclinical *in vivo* models which recapitulate the disease processes. We envisage that testing gene therapies in more representative models would be essential to allow scientists to differentiate effective from ineffective therapies before their implementation in the clinical setting (Calinescu et al., 2015; Nunez et al., 2020). The extensive molecular characterization of gliomas has been instrumental in improving our understanding of glioma progression and their response to therapies. Genetic lesions in gliomas also play a critical role in modulating the TME. We believe that all these characteristics need to be closely modeled in preclinical models in order to offer a stronger footing on which to base the development and implementation of gene therapy mediated clinical trials.

As of yet, despite showing promise in the preclinical setting, different innovative therapies have been failed to show efficacy in Phase III clinical trials for GBM. The failure of these treatments can be attributed to tumor heterogeneity, tumor immune escape, and development of resistance to the therapy, the presence of the BBB, anatomical location, GBM invasiveness and immune suppressive TME. Gene therapy approaches that rely on the transduction of most of the tumor cells to be effective, can encounter unsurmountable challenges, due to the low transduction efficacy of currently available delivery platforms. This could be overcome by the use of convection enhanced delivery to achieved widespread transduction, manual delivery at multiple tumor locations and/or combination with immune stimulatory approaches which would rely of tumor antigen specific T cells to eradicate any remaining tumor cells.

In addition, the limitations of OV include limited replication capacity of OV after a few replicative cycles, the lack of widespread distribution of the oncology viruses throughout the tumor mass. Also the immune system of the host, may curtail replicative potential of the oncolytic viruses. As the idea of gene therapy gained hold, an ideal vector system quest began. Searching the database ClinicalTrials.gov for “gene therapy/transfer and the viral delivery system,” adenovirus returns 69 studies, adeno-associated virus 41, herpes simplex virus 8, retro virus 61 and lentivirus 20 trials and plasmid delivery returns 19 studies. Currently, the use of AAV and lentiviral vectors is on the rise, while adenoviral vectors appear stable over time. Different viral vectors can be engineered to selectively replicate and kill tumor cells. In spite to the demonstrated safety of different viral and non-viral vector administration to glioma patients, gene therapy still needs to prove its potential as a valuable therapeutic tool for the treatment of gliomas. It has recently become apparent that there is a need for combinatorial treatments in order to elicit higher therapeutic efficacy and better outcomes in the clinical arena. Combinatorial immune-gene therapies offer promising approaches for improving patient survival in GBM. Considering the numerous therapeutic approaches developed, the several possible targets, the improved current SOC and alternative dosing regimens and delivery routes, the number of potential combinations has increased exponentially. Several combinatorial approaches are today under clinical trials.

In this respect, results from a Phase I clinical trial in which anti-PD-L1 was administered before and after GBM resection, demonstrated the importance of the selection of the starting point of the treatment. Moreover, as drug penetration in the brain is an issue for GBM treatment, different ways of administering these agents are being assessed and, so far, intracranial delivery, though invasive, has demonstrated to be the most efficient in several approaches. Nanoparticles have emerged as a new and safe method for the delivery of agents targeting brain tumors and preclinical results are encouraging. It would be interesting to test the efficacy of these particles for the delivery of immune-stimulatory agents in the clinical setting. Finally, there is an urgent need for increased translational research and novel clinical trials to determine the potential efficacy of these novel therapies in glioma patients.

## Author Contributions

KB, FN, SH, BLM, SVF, SC, JY, MSA, AA, AC, MLV, MC, PL, and MGC wrote the manuscript with overall guidance, revisions, and edits from PRL and MGC. All authors read and approved the final version of the manuscript.

## Conflict of Interest

The authors declare that the research was conducted in the absence of any commercial or financial relationships that could be construed as a potential conflict of interest.
